# *Bifidobacterium pseudolongum*‐Derived Bile Acid from Dietary Carvacrol and Thymol Supplementation Attenuates Colitis via cGMP‐PKG‐mTORC1 Pathway

**DOI:** 10.1002/advs.202406917

**Published:** 2024-09-23

**Authors:** Ke Zhang, Yangbin Xu, Yining Zheng, Ting Zhang, Yujiang Wu, Yiting Yan, Yu Lei, Xi Cao, Xiaolong Wang, Frances Yan, Zhaomin Lei, Daniel Brugger, Yulin Chen, Lu Deng, Yuxin Yang

**Affiliations:** ^1^ College of Animal Science and Technology Northwest A&F University Yangling 712100 China; ^2^ Institute of Animal Sciences Tibet Academy of Agricultural and Animal Husbandry Sciences Lhasa 850009 China; ^3^ Novus International Inc Research Park Drive Saint Charles MO 63304 USA; ^4^ College of Animal Science and Technology Gansu Agricultural University Lanzhou 730070 China; ^5^ Institute of Animal Nutrition and Dietetics Vetsuisse‐Faculty University of Zurich Zurich 8057 Switzerland

**Keywords:** 12‐ketodeoxycholic acid, bile acid, gut microbiota, inflammatory bowel disease, mTOR

## Abstract

Carvacrol and thymol (CAT) have been widely recognized for their antimicrobial and anti‐inflammatory properties, yet their specific effects on colitis and the mechanisms involved remain insufficiently understood. This study establishes a causative link between CAT administration and colitis mitigation, primarily through the enhancement of *Bifidobacterium pseudolongum* abundance in the colon. This increase promotes the production of secondary bile acids, particularly hyodeoxycholic acid (HDCA) and 12‐ketodeoxycholic acid (12‐KCAC), which exert anti‐inflammatory effects. Notably, CAT does not alleviate colitis symptoms in germ‐free mice, indicating the necessity of gut microbiota. This research uncovers a novel regulatory mechanism where HDCA and 12‐KCAC inhibit colonic inflammation by reducing the expression of transmembrane guanylate cyclase 1A in the colonic epithelium. This downregulation elevates intracellular Ca^2+^ and cGMP levels, activating protein kinase G (PKG). Activated PKG subsequently suppresses the mTOR signaling pathway, thereby ameliorating dextran sulfate sodium (DSS)‐induced colonic damage. These findings highlight potential metabolites and therapeutic targets for preventing and treating colitis. *Bifidobacterium pseudolongum*, HDCA, and 12‐KCAC emerge as promising candidates for therapeutic interventions in colitis and related disorders characterized by impaired tight junction function.

## Graphical abstract

Carvacrol and thymol mitigate colitis by boosting *Bifidobacterium pseudolongum* abundance, promoting secondary bile acid synthesis, particularly hyodeoxycholic acid, and 12‐ketodeoxycholic acid. These bile acids inhibit colonic inflammation by downregulating transmembrane guanylate cyclase 1A, elevating intracellular Ca^2+^ and cGMP levels, and suppressing the mTOR signaling pathway. Findings reveal promising therapeutic targets for colitis prevention and treatment.

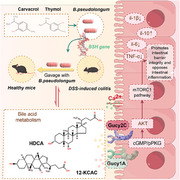

## 1 Introduction

Inflammatory bowel disease (IBD), encompassing Crohn's disease and ulcerative colitis, represents a globally recognized condition characterized by chronic immune‐mediated intestinal inflammation.^[^ ^1^ ^]^ The etiology of IBD is multifactorial, with a confluence of genetic predisposition, dietary habits, and antibiotic usage being significant contributors.^[^ ^2^ ^]^ Over the years, alterations in the gut microbiota have been implicated in the onset of chronic intestinal inflammation.^[^ ^3^ ^]^ In the majority of colitis models, the gut microbiome emerges as an indispensable factor in disease pathogenesis.^[^ ^4^ ^]^ This has propelled investigations into the metabolic processes of the intestinal microbiota and their mechanistic roles in IBD progression. Significantly, the association between microbial bile acid metabolism and IBD has been repeatedly substantiated.^[^ ^5^, ^6^, ^7^ ^]^ Microorganisms harboring bile salt hydrolase (BSH) enzymes can convert primary bile acids to secondary bile acids within the intestines, synergistically ameliorating IBD progression.^[^ ^8^, ^9^ ^]^ Our previous studies have further demonstrated that pivotal bile acid metabolites such as α‐muricholic acid (α‐MCA), hyodeoxycholic acid (HDCA), and isolithocholic acid (isoLCA) indirectly inhibit the differentiation of Th17 cells and modulate the IL‐17 signaling pathway. This modulation subsequently regulates the colonic NF‐κB and mitogen‐activated protein kinase signaling pathways, thus mitigating colitis development.^[^ ^10^ ^]^ However, a comprehensive understanding of the mechanistic interplay between these crucial microbial‐bile acid‐IBD associations remains elusive. Particularly, insights into how bile acids influence epithelial repair and intestinal mucosal healing are scant. Consequently, there is an imperative need to systematically elucidate the roles of key bile acids and their interactions with the gut microbiota in IBD therapeutics, with potential implications for therapeutic interventions. Additionally, Gucy1A and Gucy2C are two members of the guanylyl cyclase family, encoding guanylyl cyclase 1 (GC1) and guanylyl cyclase C (GC‐C), respectively. GC‐C, encoded by Gucy2C, is primarily expressed in intestinal epithelial cells,^[^ ^11^ ^]^ where it responds to enterotoxins and endogenous ligands such as guanylin and uroguanylin.^[^ ^12^ ^]^ It plays a critical role in regulating intestinal fluid and electrolyte secretion, maintaining water and salt balance, and contributing to epithelial cell proliferation and differentiation.^[^ ^13^ ^]^ However, it remains unclear whether dysregulation of the GC1 signaling pathway is associated with the onset of IBD. The potential for key bile acids to stimulate the GC1/cGMP pathway to modulate intestinal epithelial barrier function, and its interaction with Gucy2C, represents a significant knowledge gap in our understanding of IBD pathogenesis.

Carvacrol and thymol (CAT), the primary active chemical constituents of plants such as oregano and thyme,^[^ ^14^ ^]^ exhibit notable antimicrobial, antioxidant, and antiviral activities.^[^ ^15^ ^]^ In recent years, these compounds have found widespread application in food products and nutritional supplements.^[^ ^16^ ^]^ The antimicrobial action of thymol and carvacrol predominantly involves the disruption of bacterial cell membranes, reduction in biofilm formation, inhibition of microbial motility, and suppression of microbial ATPase activity.^[^ ^14^ ^]^ When combined, these compounds manifest enhanced synergistic effects in modulating immune activity within the organism. Specifically, the phenolic components of these compounds interact with dendritic cells, lymphocytes, regulatory Treg cells, macrophages, and neutrophils, thereby engaging with the immune system.^[^ ^17^ ^]^ While thymol and carvacrol have demonstrated promising anti‐inflammatory and therapeutic effects in animal models of necrotizing enterocolitis induced by pathogenic *Escherichia coli* and *Clostridium perfringens*, current research has not precisely identified the molecular targets of these compounds.^[^ ^18^ ^]^ Particularly, the lingering uncertainty regarding whether thymol and carvacrol mediate the immune‐regulatory properties through interactions with key gut microbiota and their metabolites, thereby influencing intestinal epithelial function, remains a significant puzzle. This uncertainty has raised concerns regarding the application of thymol and carvacrol in food and pharmaceuticals.

In the present study, we utilized dextran sulfate sodium (DSS) to induce colitis in mice, to establish a murine model of colitis to study potential ameliorating effects of CAT. Intriguingly, CAT did not ameliorate the onset of DSS‐induced colitis in germ‐free mice. However, in specific pathogen‐free mice, these compounds demonstrated a pronounced effect in promoting the abundance of *Bifidobacterium pseudolongum* (*B. pseudolongum*), consequently exhibiting a potent inhibitory effect on colitis progression. Through metabolomic analysis, we substantiated that pivotal metabolites of *B. pseudolongum*, namely HDCA and 12‐ketolithocholic acid (12‐KCAc), suppressed the expression of colonic epithelial guanylate cyclase 1A (Gucy1A), thereby mitigating the onset of DSS‐induced colitis. Our findings introduce a promising candidate bile acid‐based therapeutic agent, potentially suitable for oral administration, aimed at both the prophylaxis and treatment of colitis.

## 2 Results

### 2.1 Oral CAT Administration Alleviated DSS‑induced Colitis

**Figure 1:**
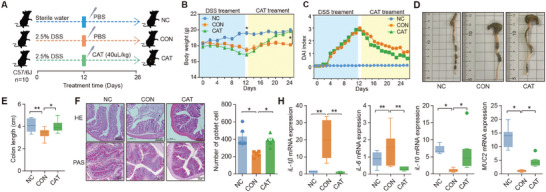
Oral CAT administration alleviated DSS‑induced colitis. A) Mice (male, *n * =  10 per group) were treated with 2.5% DSS in the presence or absence of CAT (40 µL kg^−1^ d^−1^) for 14 consecutive days. B) The body weight changes during the experiments were monitored. Mean ± SEM from three independent experiments. C) The DAI index changes during the experiments were monitored. D) Colon tissues were isolated on the last day of the experiment. A representative photograph of colon tissue from each group is provided, and the colon length was recorded E). F) The histological analysis of mouse colon tissue was performed by H&E, and alcian blue staining. Scale bar  =  200 µm. The number of goblet cells of colon tissue was evaluated (*n* = 5). H) The mRNA level of *IL‐1β*, *IL‐6*, *IL‐10*, and *MUC2* in the mouse colon was assessed by qPCR analysis (*n* = 10). ^*^ *p* < 0.05, ^**^ *p* < 0.01, ^***^ *p *< 0.001. Statistical significance was determined using one‐way ANOVA, followed by the Tukey test.

To examine the therapeutic efficacy of CAT in DSS‐induced colitis, experimental colitis was induced in mice via continuous administration of 2.5% DSS in drinking water for 12 days until a significant discrepancy in body weight among the mice was observed (*p* < 0.05; **Figure** **1**B). Subsequently, all mice were orally administered CAT at the optimal dose determined from previous studies (40 µL kg^−1^) on a daily basis. Compared to the Con group, oral CAT intervention significantly alleviated DSS‐induced colitis, as evidenced by a marked reduction in disease activity index (DAI) scores, including weight loss and fecal consistency, as well as reduced weight loss and colon shortening (*p* < 0.05; Figure 1C–E). Histological analysis further revealed a notable attenuation of colonic inflammation, mucosal damage, and overall histological scores in the CAT group compared to the Con group. Additionally, alcian blue staining demonstrated a significant increase in the number of goblet cells in colonic tissues (*p* < 0.05; Figure 1F). Gene expression analysis in colonic tissues revealed a significant decrease in pro‐inflammatory cytokines *IL‐1β* and *IL‐6*, along with a significant increase in the expression of the anti‐inflammatory cytokine *IL‐10*. Furthermore, the expression of colonic mucin *MUC2* was significantly upregulated (*p* < 0.05; Figure 1H). Collectively, these findings demonstrate that oral administration of CAT ameliorates clinical symptoms and colonic damage in experimental colitis.

### 2.2 CAT Alleviated DSS‑induced Colitis in a Gut Microbiota‐Dependent Manner

In the present study, we observed significant interindividual variability in the recovery of mice during the treatment of DSS‐induced colitis with CAT. This led us to hypothesize that the alleviation of clinical symptoms and colonic damage by CAT might be mediated through the modulation of the gut microbiota. To investigate this further, we generated a pseudo‐germ‐free mouse model by treating mice with a combination of antibiotics, and subsequently performed CAT treatment following the same protocol (Figure S1A, Supporting Information). Interestingly, the previously observed improvements in clinical symptoms following CAT treatment were completely abolished in the antibiotic‐treated mice, including changes in body weight, DAI scores, colon length, tissue inflammation, expression of pro‐inflammatory and anti‐inflammatory cytokine genes, and *MUC2* gene expression (*p* > 0.05; Figure S1B–H, Supporting Information). Quantitative PCR and 16S rRNA gene sequencing analysis revealed that the gut microbiota diversity and composition of DSS‐induced colitis mice were not altered by oral CAT treatment after the clearance of intestinal bacteria by the antibiotic treatment (Figure S1I–K, Supporting Information). Notably, the abundance of *Lactobacilli* and *Bifidobacteria* was almost undetectable after antibiotic treatment (Figure S1L, Supporting Information). Taken together, these results further support the role of the gut microbiota in mediating the improvement of clinical symptoms and colonic damage by CAT.

**Figure 2:**
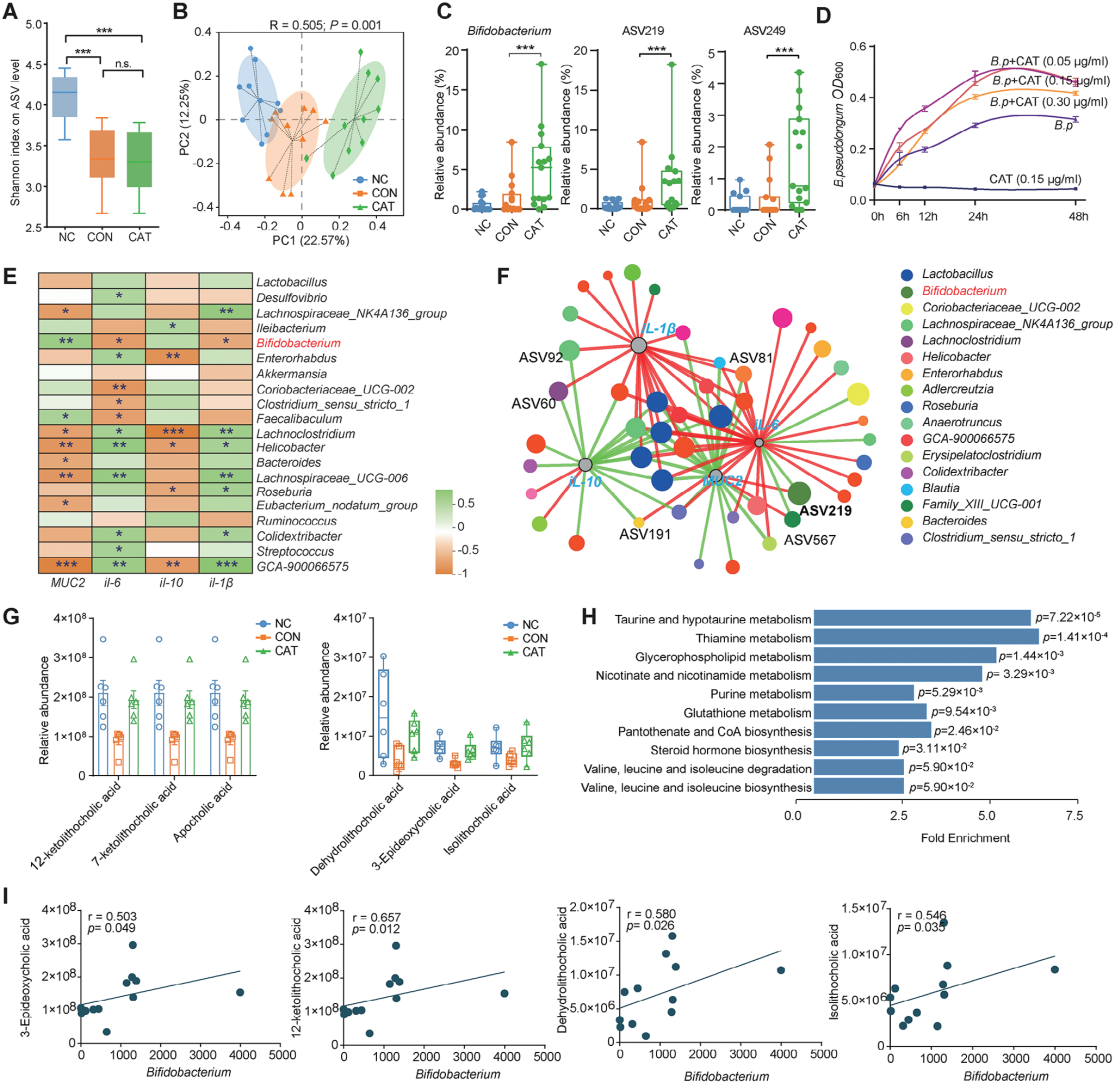
CAT alleviated DSS‑induced colitis in a gut microbiota–dependent manner A) α‐diversity upon oral therapy represented by the Shannon index (*n*  =  10 per group). Statistical significance was determined using one‐way ANOVA, followed by the Tukey test. ^*^ *p* < 0.05, ^**^ *p* < 0.01, ^***^ *p *< 0.001. B) PCoA plot based on ASV abundances (*n * =  10 per group). The colors of the symbols indicate different treatment groups, respectively. An analysis of similarity (ANOSIM) was used to assess the dissimilarity of Bray–Curtis. C) The relative abundance of key microbes of *Bifidobacterium*, ASV219 and ASV249. Statistical significance was determined using a pairwise Wilcoxon test and two‐tailed Fisher's test with FDR correction (*n*  =  10 per group). ^*^ *p* < 0.05, ^**^ *p* < 0.01, ^***^ *p *< 0.001. D) Inoculation of *Bifidobacterium pseudolongum* RU224 strains was conducted in BSM medium supplemented with CAT at concentrations of 0.05, 0.15, and 0.30 µg mL^−1^. The optical density (OD) of the strains was measured at 0, 6, 12, 24, and 48 h to generate growth curves under different CAT concentrations. The BP group served as a positive control without CAT supplementation. The negative control consisted of BSM medium supplemented with only 0.15 µg mL^−1^ of CAT but without inoculated strains. E) Spearman correlation analysis was conducted between the abundance of selected colonic differential microbial genera and the expression levels of inflammatory cytokines in colonic tissues. Additionally, correlation networks were constructed among important ASVs and the expression levels of inflammatory cytokines associated with colonic tissue inflammation F). Green lines represent negative correlations between two variables, while red lines represent positive correlations, with an absolute Spearman's correlation coefficient (|r|) greater than 0.6 and *p*‐value < 0.05, ^*^ *p* < 0.05, ^**^ *p* < 0.01, ^***^ *p *< 0.001. G) Differential bile acids and their abundance obtained through comprehensive targeted metabolomics screening were identified. H) Enriched metabolic pathways significantly altered by CAT treatment compared to the DSS group were identified using Gene Set Enrichment Analysis (GSEA). I) Spearman correlation analysis was employed to investigate the correlation between *Bifidobacterium* and key differential metabolites. Correlation coefficients greater than 0.5 with a *p*‐value <0.05 were considered significant.

Therefore, to further identify key microbiota that mediate the beneficial effects of CAT on clinical symptoms and colonic damage in colitis, we performed 16S rRNA gene sequencing analysis on the colonic contents of SPF mice treated with CAT. The results revealed that the α‐diversity at the ASV level in colitis mice was not affected by oral CAT administration (**Figure** **2**A; Figure S2A, Supporting Information). Principal coordinate analysis (PCoA) based on Bray‐Curtis and unweighted UniFrac distances demonstrated a separation in the gut microbiota composition between the control group and colitis mice (ANOSIM; R = 0.585, *p* = 0.001, Figure 2B; Figure S2B, Supporting Information). Furthermore, a separation in the gut microbiota composition was observed between the CAT and Con groups (ANOSIM; R = 0.295, *p* = 0.001, Figure 2B), indicating a significant impact of oral CAT administration on the gut microbiota composition in colitis mice. At the phylum level, Firmicutes and Bacteroidota were the dominant phyla in the colonic microbiota. CAT treatment significantly reduced the abundance of Bacteroidota and increased the abundance of Actinobacteriota in colitis mice (Figure S2C, Supporting Information). At the genus level, CAT treatment significantly increased the abundance of *Turicibacter*, *Dubosiella*, and *Bifidobacterium* in colitis mice (*p* < 0.001; Figure 2C; Figure S2D, Supporting Information). Differential abundance analysis using LEfSe identified bacterial taxa with differential abundance in response to oral CAT treatment in DSS‐treated mice, including the enrichment of seven bacterial genera, including *Bifidobacterium*, and the enrichment of three other taxa, including *Lachnospiraceae_NK4A136_group*, specifically in the DSS group (Figure S3A, Supporting Information). Moreover, two ASVs belonging to the *Bifidobacterium* genus showed a significant increase in abundance in the colon of colitis mice after oral CAT administration (Figure 2C). Phylogenetic tree analysis using the sequences of ASV249 and ASV219 revealed high sequence similarity (Query Cover = 100%, Per. Ident = 99.53%, Figure S2E, Supporting Information) with *B. pseudolongum* RU224 and *B. pseudolongum* PNG‐2‐9G, respectively. Further analysis of fecal *B. pseudolongum* RU224 abundance using quantitative PCR revealed that CAT treatment significantly increased the abundance of *B. pseudolongum* RU224 in the gut depleted by DSS treatment (*p* < 0.001; Figure S2F, Supporting Information). Spearman correlation analysis further demonstrated a significant negative correlation between the abundance of *Bifidobacterium* and the expression of pro‐inflammatory cytokine genes (*r* > ‐0.5, *p* < 0. 0.05) and a significant positive correlation with *MUC2* gene expression (*r* > 0.6, *p* < 0.05, Figure 2E). Similarly, ASV219 showed a significant negative correlation with *IL‐6* gene expression in the colon (*r*> ‐0.5, *p* < 0.05; Figure 2F). Functional prediction analysis of microbiota revealed that compared to SPF mice, DSS treatment significantly upregulated the “taurine and hypotaurine metabolism pathway” in the colon of mice (*p* < 0.05; Figure S3B, Supporting Information), while pathways such as “fatty acid degradation,” “fatty acid metabolism,” “fatty acid biosynthesis,” and “pyruvate metabolism” were significantly downregulated following CAT treatment (*p* < 0.05; Figure S3B, Supporting Information). This further suggests that CAT treatment may mediate microbial involvement in modulating lipid metabolism capacity in DSS mice.

To profile the metabolic changes, widely targeted metabolomics was conducted with mouse colon content. Principal component analysis of the overall metabolite composition revealed that the Con group was separated from the CAT group (Figure S4A,B, Supporting Information). The colon metabolic profiles and metabolic pathways of orally administered CAT mice showed great differences from those of DSS mice (Figure S4C,D, Supporting Information). Notably, there was a marked elevation in the abundance of bile acids in the colon, particularly 12‐ketolithocholic acid (12‐KDCA), 7‐ketolithocholic acid, apocholic acid, dehydrolithocholic acid, 3‐Epideoxycholic acid, and isolithocholic acid (Figure 2G). Metabolite set enrichment analysis (MSEA) identified that the metabolites exhibiting significant differences were primarily enriched in the “taurine and hypotaurine metabolism” and “thiamine metabolism pathways” (*p* < 0.001, Figure 2H; Figure S4E,F, Supporting Information). Further correlation analysis revealed a significant positive association between the abundance of *B. pseudolongum* in the colon and the levels of 12‐KDCA, dehydrolithocholic acid, 3‐Epideoxycholic acid, and isolithocholic acid (r > 0.6, *p* < 0.05; Figure 2I). These comprehensive findings collectively suggest that oral CAT treatment modulates the composition and potential functions of the gut microbiota in colitis mice, with *B. pseudolongum* and bile acids potentially playing a pivotal role in alleviating DSS‐induced colitis.

### 2.3 CAT‐Mediated Enrichment of *Bifidobacterium pseudolongum* Alleviated DSS‑induced Colitis

To determine whether the addition of CAT promotes the proliferation of *B. pseudolongum* in vitro, we isolated the crucial strain *B. pseudolongum RU224* (BP) from the colon of CAT‐treated colitis mice using BSM medium. Subsequently, different concentrations of CAT were added to the BSM medium, and the results indicated that, under in vitro conditions, the addition of CAT rapidly promoted the proliferation of *B. pseudolongum RU224* (Figure 2D). Further analysis through whole‐genome sequencing revealed the presence of the choloylglycine hydrolase gene in the genome of *B. pseudolongum* RU224 (Figure S5A, Supporting Information), participating in the primary bile acid biosynthesis (KO 00120) and secondary bile acid biosynthesis pathways (KO 00121). Key genes involved in lipid metabolism, such as *pldb*, *galA*, *gpsA*, *lacZ*, *bccA*, *fas*, *adhE*, *mgdA*, *tesB*, *srfJ*, *fadD*, *cdsA*, *pgsA*, *BSH*, *glxK*, and *plsC*, were identified in the genome of *B. pseudolongum* RU224 (Figure S5B, Supporting Information).

**Figure 3:**
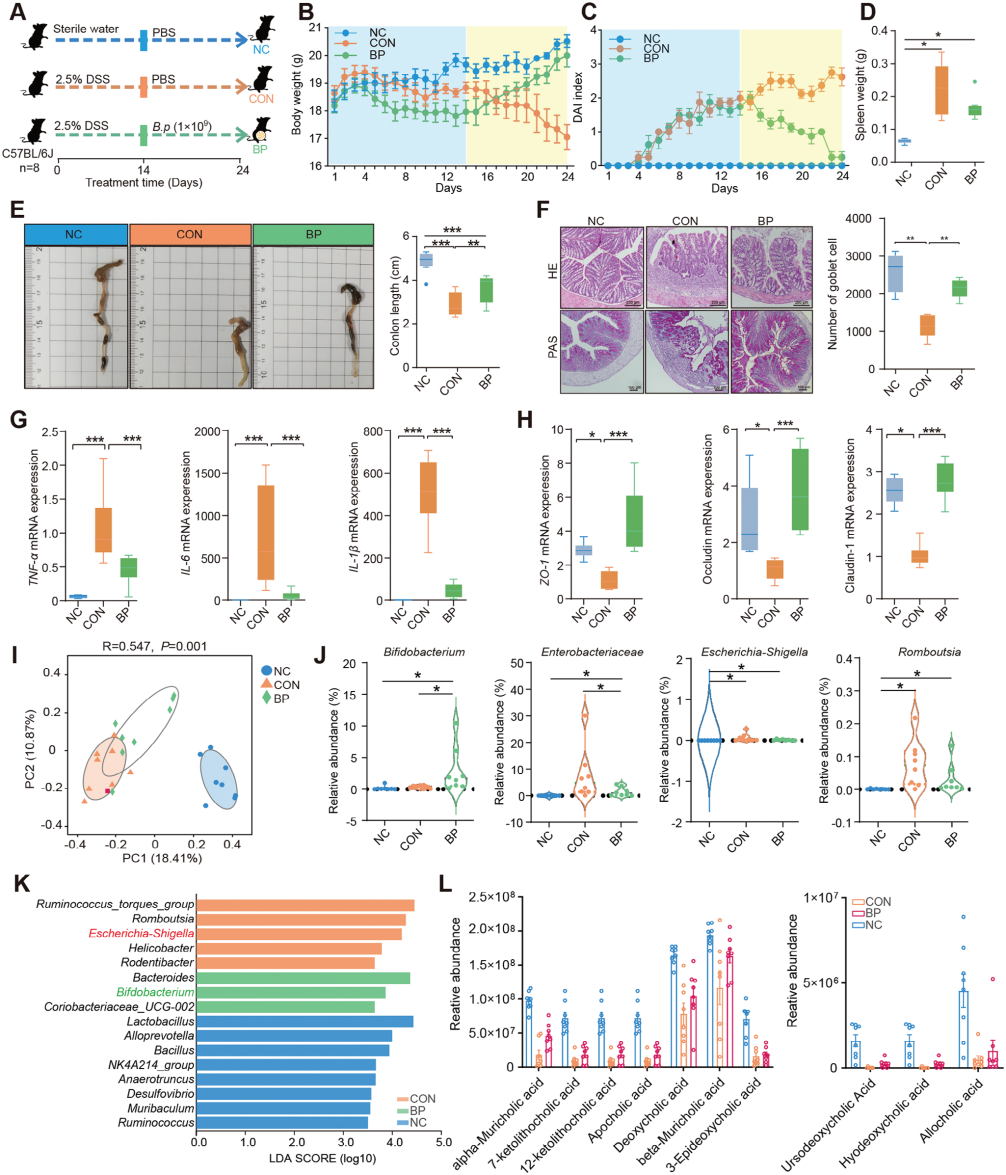
*Bifidobacterium pseudolongum* promotes the generation of secondary bile acids and alleviates DSS‐induced colitis. A) Male mice (*n* = 8 per group) were treated with 2.5% DSS in the presence or absence of BP (1 × 10^9^ CFU/d) for 12 consecutive days. B) Monitoring of body weight changes during the experiments. Mean ± SEM from three independent experiments. C) Monitoring of changes in the Disease Activity Index (DAI) during the experiments. D) Evaluation of spleen weight. Statistical significance was determined using one‐way ANOVA, followed by Tukey's test. ^*^ *p* < 0.05. E) Colon tissues were isolated on the last day of the experiment. Representative photographs of colon tissue from each group were provided, and colon length was recorded. Statistical significance was determined using one‐way ANOVA, followed by Tukey's test. ^*^ *p* < 0.05, ^**^ *p* < 0.01, ^***^ *p* < 0.001. F) Histological analysis of mouse colon tissue was performed using H&E and alcian blue staining. Scale bar = 200 µm. The number of goblet cells in colon tissue was evaluated (*n* = 5). G) qPCR analysis of mRNA levels of IL‐1β, IL‐6, IL‐10, and MUC2 in mouse colon tissue (*n* = 10). ^*^ *p* < 0.05, ^**^ *p* < 0.01, ^***^ *p* < 0.001. Statistical significance was determined using one‐way ANOVA, followed by Tukey's test. H) qPCR analysis of mRNA levels of *ZO‐1*, *Occludin*, and *Claudin‐1* in mouse colon tissue (*n* = 8). ^*^ *p* < 0.05, ^**^ *p* < 0.01, ^***^ *p* < 0.001. Statistical significance was determined using one‐way ANOVA, followed by Tukey's test. I) Principal Coordinate Analysis (PCoA) plot based on ASV abundances (*n* = 8 per group). The colors of symbols indicate different treatment groups, respectively. Analysis of Similarity (ANOSIM) was used to assess the dissimilarity of Bray–Curtis. J) Relative abundance of key microbes including *Bifidobacterium*, *Enterobacteriaceae*, *Escherichia‐Shigella*, and *Romboutsia*. Statistical significance was determined using pairwise Wilcoxon test and two‐tailed Fisher's test with FDR correction (*n* = 8 per group). ^*^ *p* < 0.05. K) Selection of differential microbial genera using LEfSe analysis among the three groups. Linear Discriminant Analysis (LDA) scores showed significant enrichment for taxa (*p* < 0.05 and |LDA| >3) when all samples were treated as independent. L) Identification of differential bile acids and their abundance through comprehensive targeted metabolomics screening.

Further evaluation of the therapeutic effects of *B. pseudolongum* RU224 on DSS‐induced colitis (**Figure** **3**A). The oral administration significantly alleviated DSS‐induced colitis, as evidenced by a marked reduction in the DAI, decreased weight loss, and alleviated colon shortening (*p* < 0.05; Figure 3B–E). Histological analysis further revealed a substantial reduction in colonic inflammation, mucosal damage, and overall histological scores in the BP group compared to the Con group (Figure 3F). Alcian blue staining demonstrated a significant increase in goblet cell numbers in colonic tissues (*p* < 0.01; Figure 3F). Gene expression analysis indicated a significant decrease in the expression levels of pro‐inflammatory cytokines *TNF‐α*, *IL‐1β*, and *IL‐6* in colonic tissues of the BP group (*p* < 0.001; Figure 3G). Additionally, the expression levels of colonic barrier proteins (*ZO‐1*, *Occludin*, and *Claudin‐1*) were significantly upregulated in the BP group compared with Con group (*p* < 0.001; Figure 3H). These results further confirm that oral administration of live *B. pseudolongum* RU224 effectively mitigates DSS‐induced colitis.

To investigate the impact of oral administration of live *B. pseudolongum* RU224 on the gut microbiota composition of DSS‐induced colitis mice, 16S rRNA gene sequencing revealed significant alterations in the colonic microbiota composition in the BP group (Figure 3I). Specifically, there was an increase in the abundance of *Bifidobacterium* and *Bacteroides* genera, while the abundance of *Enterobacteriaceae* was decreased in the colon (*p* < 0.05; Figure 3J,K). Similarly, the BP group exhibited an increase in the abundance of bile acids, including 7‐ketolithocholic acid, 12‐KCAC, alpha‐muricholic acid, ursodeoxycholic acid, and other ten primary and secondary bile acids (Figure 3L). These results further confirm that *B. pseudolongum* RU224 can improve the composition of the colonic microbiota, promote the excretion of secondary bile acids, and alleviate the occurrence of DSS‐induced colitis.

### 2.4 *B. pseudolongum* Activates cGMP‐PKG Signaling Pathway to Inhibit DSS‑induced Colitis

To delineate the molecular basis underlying the regulation of DSS‐induced colitis by BP, we performed RNA‐Seq analysis on colonic samples from BP‐treated mice. Transcriptomic profiling of BP‐treated colonic tissue revealed a divergence from that of DSS‐exposed mice (Figure S6A, Supporting Information), with 280 up‐regulated and 432 down‐regulated genes in BP‐treated mice compared to their DSS counterparts (Figure S6A, Supporting Information). Notably, a significant downregulation in the expression of pro‐inflammatory genes was observed in the BP‐treated group compared to DSS‐treated mice, including but not limited to *Ccl4*, *Ccl3*, *Cxcl2*, *Cxcl3*, *Il1f9*, *Il20ra*, and *Il11* (*p* < 0.001; Figure S6B, Supporting Information). In addition, genes associated with the transport of various substrates across biological membranes, such as *Slc22a27*, *Slc16a11*, and *Slc22a1*, showed significant upregulation in the BP‐treated group (*p* < 0.001; Figure S6C, Supporting Information). KEGG pathway analysis revealed that DEGs in the BP‐treated group were predominantly enriched in classical inflammatory pathways, including the “IL‐17 signaling pathway,” “inflammatory mediator regulation of TRP channels,” and “cytokine‐cytokine receptor interaction” (Figure S6D, Supporting Information).

**Figure 4:**
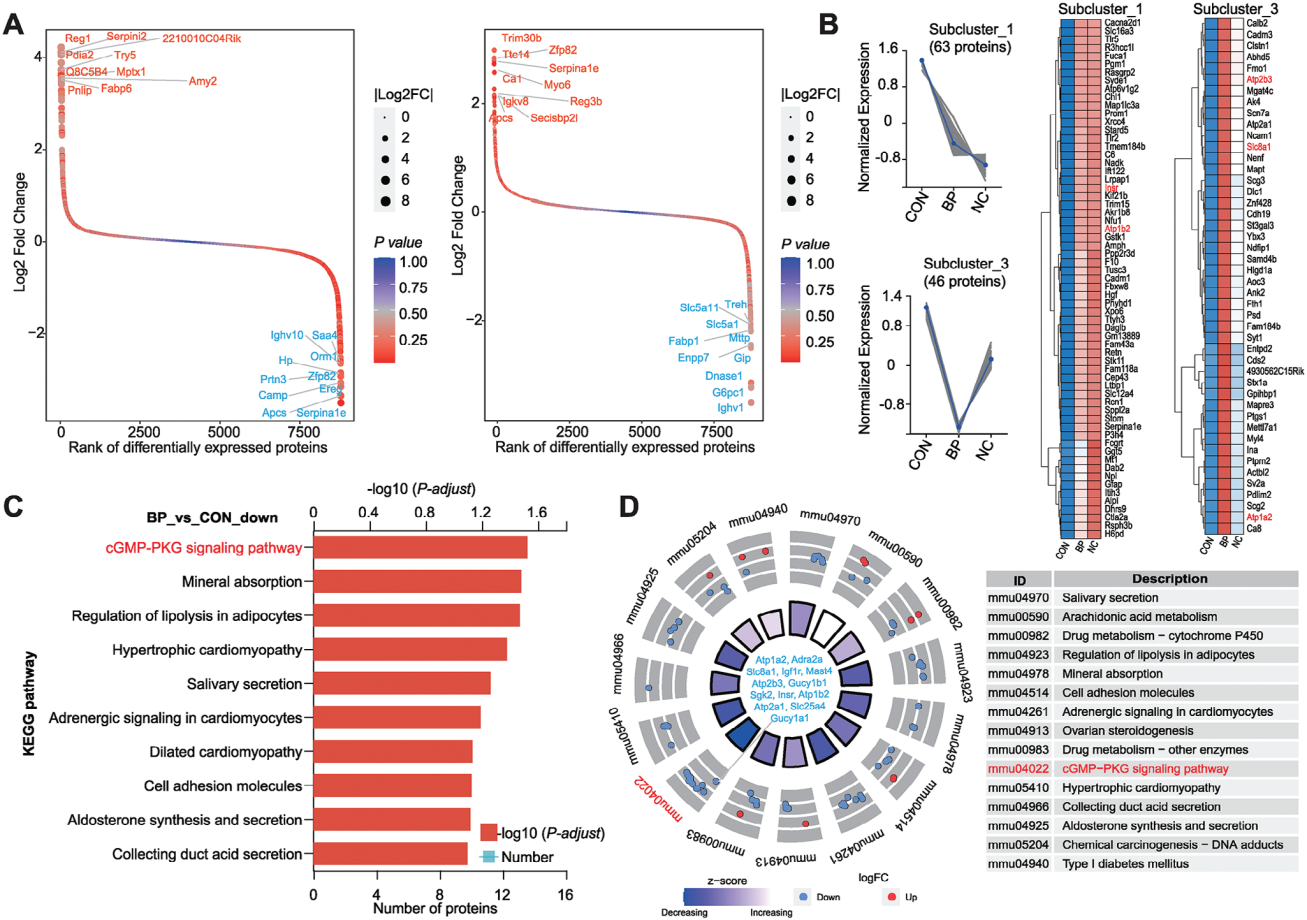
*B. pseudolongum* activates the cGMP‐PKG signaling pathway to inhibit DSS‐induced colitis. A) Volcano plots depicting protein differential expression between healthy and DSS individuals, and between DSS and BP treatment individuals in the colon (*p* < 0.05, two‐sided Student's *t*‐test). Red and blue dots represent upregulated and downregulated proteins, respectively. B) Unsupervised k‐means clustering analysis of BP treatment proteins. The expression patterns of BP treatment‐related proteins in distinct clusters are shown. The mainly differentially expressed proteins in each subcluster are shown in the right panel. C) Metabolic pathways enriched by differentially expressed proteins based on KEGG analysis between BP and CON treatment groups. D) Enrichment circle plot revealing differential metabolic pathways induced by BP treatment and the differential proteins involved in these pathways. Upregulated and downregulated proteins are represented in red and blue colors, respectively. The inner circle ranges from purple to red, representing the z‐score values used to estimate biological processes that may be activated or inhibited.

To further elucidate the key target proteins modulated by BP in the regulation of DSS‐induced colitis at the protein level, we performed proteomic sequencing on colonic samples from BP‐treated mice. Compared to SPF mice, the expression of serine protease inhibitor (Serpina1e) and amyloid P component (APCS), hallmark proteins of colitis, was upregulated more than fourfold in DSS‐treated mice (**Figure** **4**A), whereas BP treatment led to a significant downregulation of these two hallmark proteins by more than twofold (*p* < 0.001; Figure 4A). Using clustering analysis, we identified two protein clusters of 63 and 46 differential proteins, respectively, that were significantly downregulated in the BP‐treated group compared to the DSS group (Figure 4B). Of particular note is the significant downregulation of target proteins associated with the maintenance of intracellular and extracellular ion concentrations, including Atp1a2, Adra2a, Atp2b3, Atp1b2, Atp2a1, guanylate cyclase proteins Gucy1a1 and Gucy1b1, in the BP group (*p* < 0.001; Figure 4B). Importantly, these downregulated proteins are mainly enriched in the cGMP‐PKG signaling pathway (*p* < 0.001; Figure 4C,D). Taken together, these findings suggest that *B. pseudolongum* RU224 colonization can modulate the cGMP signaling axis in the colonic epithelium, thereby exerting a pronounced anti‐inflammatory effect.

### 2.5 HDCA and 12‐KCAC as Key Metabolites Inhibit Inflammation in Intestinal Epithelial Cells

**Figure 5:**
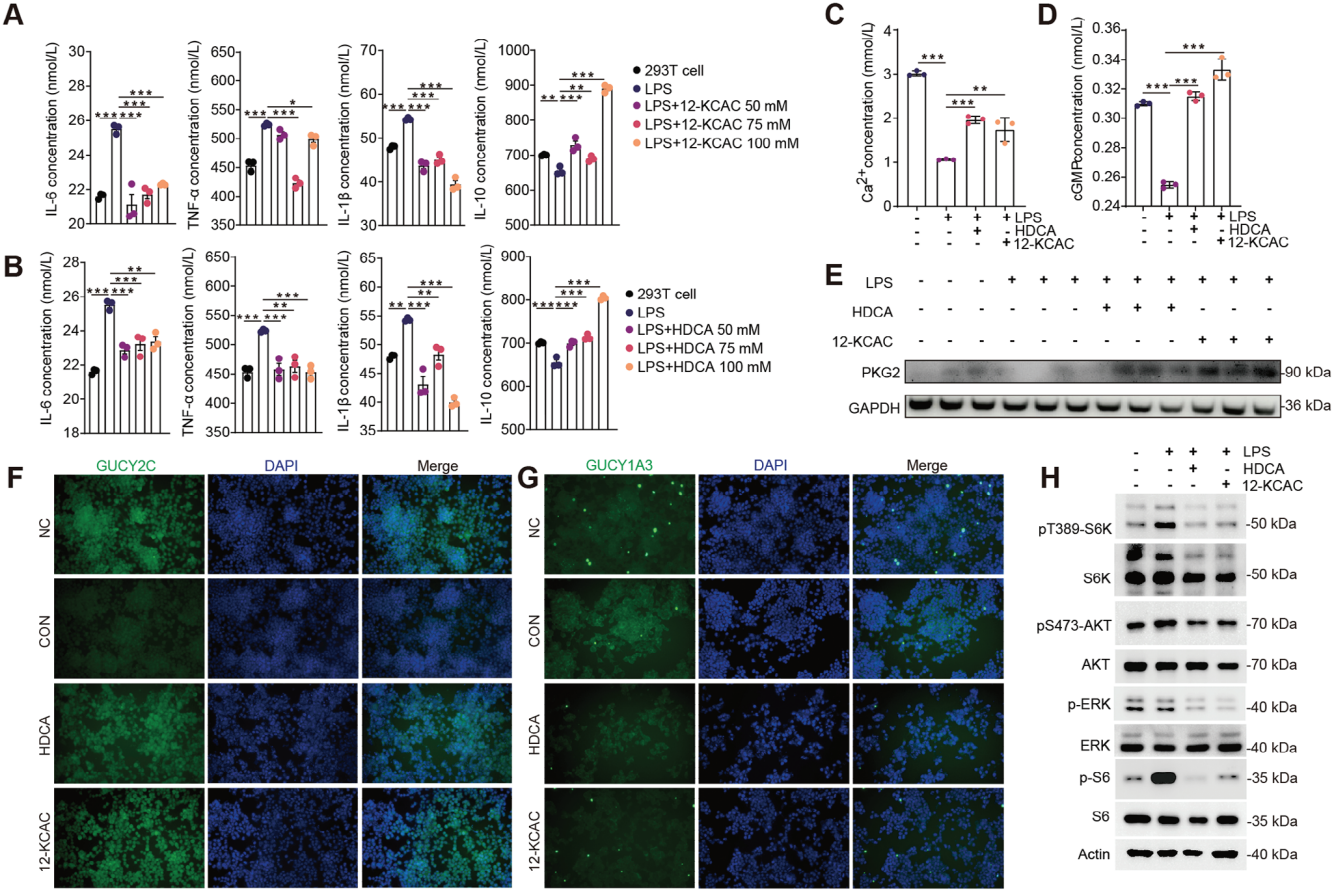
HDCA and 12‐KCAC as key metabolites inhibit inflammation in intestinal epithelial cells. A,B) Changes in the concentrations of pro‐inflammatory and anti‐inflammatory cytokines in LPS‐induced 293T inflammatory cell model upon treatment with HDCA and 12‐KCAC. ^*^ *p* < 0.05, ^**^ *p* < 0.01, ^***^ *p* < 0.001. Statistical significance was determined using one‐way ANOVA, followed by Tukey's test. C,D) Levels of Ca^2+^ and cGMP in the supernatant of LPS‐induced 293T inflammatory cells treated with HDCA and 12‐KCAC. E) Assessment of the levels of PKG2 n LPS‐induced 293T inflammatory cell model upon treatment with HDCA and 12‐KCAC by western blot analysis. F) Assessment of the levels of Gucy2C and Gucy1A3 LPS‐induced 293T inflammatory cell model upon treatment with HDCA and 12‐KCAC by immunofluorescence analysis. H) Assessment of the levels of pT389‐S6K, pS473‐AKT, p‐ERK, and p‐S6 in 293T cells by western blot analysis. ^*^ *p* < 0.05, ^**^ *p* < 0.01, ^***^ *p* < 0.001. Statistical significance was determined using one‐way ANOVA, followed by Tukey's test.

To further validate the anti‐inflammatory effects of the ten key bile acids selected by the omics analysis, we used LPS‐treated 293T cells to establish an inflammatory cell model. These cells were co‐incubated with each of the ten key bile acids. Our results show that compared to the other eight bile acids (Figure S7, Supporting Information), HDCA and 12‐KCAC significantly reduced the levels of the pro‐inflammatory cytokines IL‐6, IL‐1β, and TNF‐α, while increasing the levels of the anti‐inflammatory cytokine IL‐10 in 293T cells (*p* < 0.05; **Figure** **5**A,B; Figure S8, Supporting Information). To further confirm whether *B. pseudolongum* RU224 causally promotes 12‐KCAC and HDCA production, we performed in vitro fermentation using bile salt medium (BSM) supplemented with either inactivated or fresh feces, followed by inoculation with live or heat‐killed *B. pseudolongum* RU224. The results demonstrated that inoculation with live *B. pseudolongum* RU224 significantly increased the concentrations of 12‐KCAC and HDCA in the bacterial culture (*p* < 0.05; Figure S9, Supporting Information), while inoculation with heat‐killed *B. pseudolongum* RU224 did not result in a significant increase in these bile acids (*p* > 0.05; Figure S9, Supporting Information). These findings further support that *B. pseudolongum* RU224 exerts anti‐inflammatory effects by metabolizing certain lipids in the gut into 12‐KCAC and HDCA through its functional genes.

Extensive previous research has established the role of the Gucy2C‐cGMP signaling axis in counteracting intestinal epithelial injury and tumors.^[^ ^19^, ^20^ ^]^ To further test whether HDCA and 12‐KCAC inhibit the guanylate cyclase homolog Gucy1A and activate the Gucy2C transmembrane receptor to counteract intestinal injury (Figure 5F), we found that stimulation with HDCA and 12‐KCAC significantly inhibited the protein expression of the Gucy1A transmembrane receptor in colonic epithelial cells, while significantly activating the expression of the Gucy2C transmembrane receptor protein (*p* < 0. 05; Figure 5G). In addition, intracellular Ca^2+^ and cGMP levels were significantly increased by stimulation with HDCA and 12‐KCAC (*p* < 0.05; Figure 5C,D). Further examination of the expression levels of key proteins regulating intestinal inflammation revealed that stimulation with HDCA and 12‐KCAC significantly upregulated the expression of PKG protein (*p* < 0.05; Figure 5E). Moreover, to explore the effect of pharmacological HDCA and 12‐KCAC on mTOR pathway activation, we treated HEK293T cells with HDCA and 12‐KCAC and examined the activation status of mTOR by assessing the levels of S6K, S6, and AKT phosphorylation. The results showed that HDCA and 12‐KCAC significantly reduced the level of pS473‐AKT, pT389‐S6K, p‐ERK, and p‐S6 in HEK293T cells (Figure 5H).

**Figure 6:**
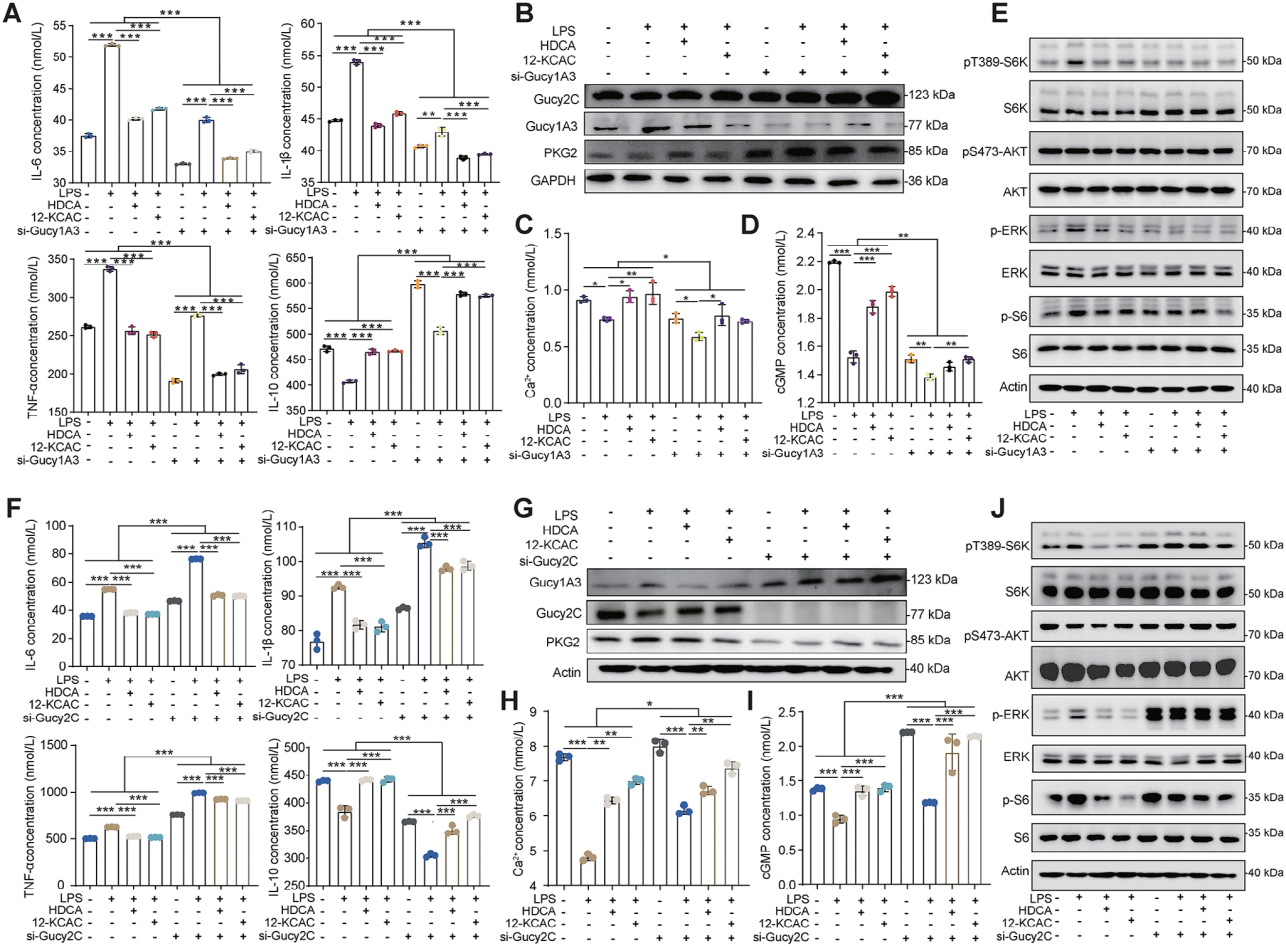
HDCA and 12‐KCAC dependence on colonic epithelial Gucy1A and Gucy2C receptor to exert anti‐inflammatory effects. A) Changes in the concentrations of pro‐inflammatory and anti‐inflammatory cytokines in 293T cells treated with HDCA and 12‐KCAC upon Gucy1A3 silencing. B) Western blot analysis of Gucy1A3, Gucy2C, and PKG2 levels in 293T cells treated with HDCA and 12‐KCAC upon Gucy1A3 silencing. C,D) Assessment of Ca^2+^ and cGMP levels in 293T cells upon Gucy1A3 silencing and treatment with HDCA and 12‐KCAC. E) Assessment of pT389‐S6K, pS473‐AKT, p‐ERK, and p‐S6 levels in 293T cells upon Gucy1A3 silencing and treatment with HDCA and 12‐KCAC by western blot analysis. ^*^ *p* < 0.05, ^**^ *p* < 0.01, ^***^ *p* < 0.001. Statistical significance was determined using one‐way ANOVA, followed by Tukey's test. F) Changes in the concentrations of pro‐inflammatory and anti‐inflammatory cytokines in 293T cells treated with HDCA and 12‐KCAC upon Gucy2C silencing. G) Western blot analysis of Gucy1A3, Gucy2C, and PKG2 levels in 293T cells treated with HDCA and 12‐KCAC upon Gucy2C silencing. H,I) Assessment of Ca^2+^ and cGMP levels in 293T cells upon Gucy2C silencing and treatment with HDCA and 12‐KCAC. J) Assessment of pT389‐S6K, pS473‐AKT, p‐ERK, and p‐S6 levels in 293T cells upon Gucy2C silencing and treatment with HDCA and 12‐KCAC by western blot analysis. ^*^ *p* < 0.05, ^**^ *p* < 0.01, ^***^ *p* < 0.001. Statistical significance was determined using one‐way ANOVA, followed by Tukey's test.

We hypothesized that HDCA and 12‐KCAC mediate mTOR inactivation through the Gucy1A and Gucy2C acceptors. To validate this hypothesis, we first used specific siRNAs to knock down Gucy1A and observed that knockdown of the Gucy1A gene led to a significant decrease in the levels of the pro‐inflammatory cytokines IL‐6, IL‐1β and TNF‐α (*p* < 0.05; **Figure** **6**A), while the levels of the anti‐inflammatory cytokine IL‐10 were significantly increased in the cells (*p* < 0.05; Figure 6A). This also resulted in the upregulation of Gucy2C and PKG2 protein expression (Figure 6B). Conversely, the levels of Ca^2+^ and cGMP in the cells were significantly reduced due to the decreased expression of the Gucy1A gene (*p* < 0.05; Figure 6C,D). Downregulation of Gucy1A reversed the HDCA‐ and 12‐KCAC‐induced inhibition of mTOR activation (Figure 6E). Second, we used specific siRNAs to knock down Gucy2C and observed that knockdown of the Gucy2C gene significantly increased the levels of pro‐inflammatory cytokines IL‐6, IL‐1β and TNF‐α (*p* < 0.05; Figure 6F), whereas the levels of anti‐inflammatory cytokine IL‐10 were significantly decreased in the cells (*p* < 0.05; Figure 6F). This also resulted in the upregulation of Gucy1A and downregulation of PKG2 protein expression (Figure 6G). Conversely, the levels of Ca^2+^ and cGMP in the cells were significantly increased due to the decreased expression of the Gucy2C gene (*p* < 0.05; Figure 6H,I). The downregulation of Gucy2C reversed the HDCA‐ and 12‐KCAC‐induced enhancement of mTOR activation (Figure 6J). These results further confirmed the important role of Gucy1A and Gucy2C in the regulation of intestinal inflammation.

### 2.6 HDCA and 12‐KCAC Inhibit Gucy1A Transmembrane Receptor in Colonic Alleviating DSS‐induced Colitis

**Figure 7:**
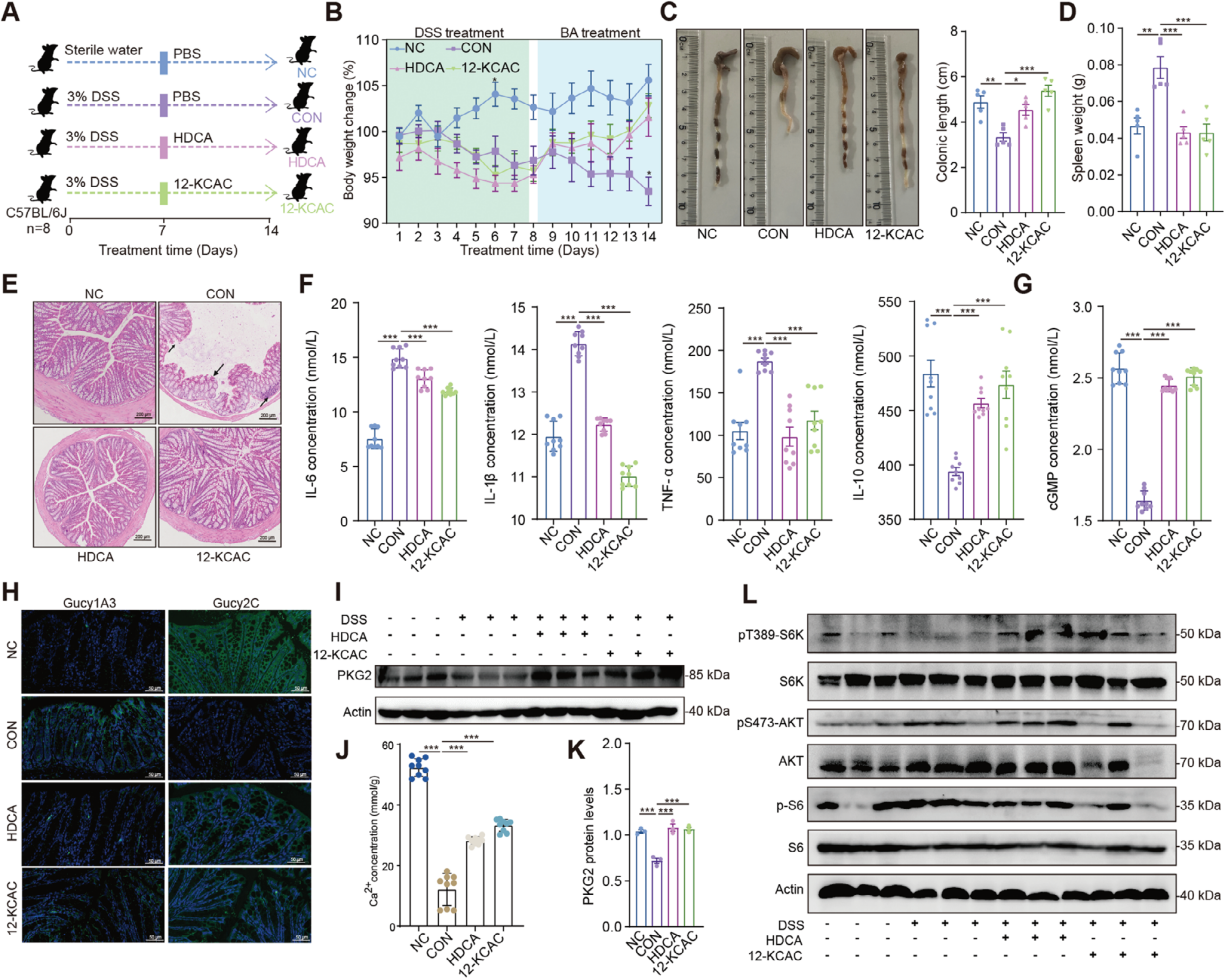
HDCA and 12‐KCAC inhibit Gucy1A transmembrane receptor in the colon to alleviate DSS‐induced colitis. A) Male mice (*n* = 8 per group) were treated with 3% DSS in the presence or absence of HDCA and 12‐KCAC (50 mg k^−1^g day^−1^) for 7 consecutive days. B) Monitoring of body weight changes during the experiments. Mean ± SEM from three independent experiments. C) Colon tissues were isolated on the last day of the experiment. A representative photograph of colon tissue from each group is provided, and colon length was recorded. D) Evaluation of spleen weight. F) Histological analysis of mouse colon tissue was performed using H&E and alcian blue staining. Scale bar = 200 µm. The number of goblet cells in colon tissue was evaluated (*n* = 5). F) Assessment of IL‐1β, IL‐6, IL‐10, and TNF‐α concentrations in mouse colon tissue. ^*^ *p* < 0.05, ^**^ *p* < 0.01, ^***^ *p* < 0.001. Statistical significance was determined using one‐way ANOVA, followed by Tukey's test. G) Levels of cGMP in the colon of DSS‐induced colitis mice treated with HDCA and 12‐KCAC. ^*^ *p* < 0.05, ^**^ *p* < 0.01, ^***^ *p* < 0.001. Statistical significance was determined using one‐way ANOVA, followed by Tukey's test. H) Immunofluorescence evaluation of Gucy1A3 and Gucy2C protein expression in the colon of DSS‐induced colitis mice treated with HDCA and 12‐KCAC. I) Assessment of PKG2 levels in the colon of DSS‐induced colitis mice treated with HDCA and 12‐KCAC by western blot analysis, and quantification of protein band grayscale values using Image J. J) Levels of Ca^2+^ in the colon of DSS‐induced colitis mice treated with HDCA and 12‐KCAC. ^*^ *p* < 0.05, ^**^ *p* < 0.01, ^***^ *p* < 0.001. Statistical significance was determined using one‐way ANOVA, followed by Tukey's test. K) Quantification of PKG2 protein band grayscale values using Image J. ^*^ *p* < 0.05, ^**^ *p* < 0.01, ^***^ *p* < 0.001. Statistical significance was determined using one‐way ANOVA, followed by Tukey's test. L) Assessment of pT389‐S6K, pS473‐AKT, p‐ERK, and p‐S6 levels in the colon of DSS‐induced colitis mice treated with HDCA and 12‐KCAC by western blot analysis.

To further validate the alleviating effects of HDCA and 12‐KCAC on DSS‐induced colitis in vivo, experimental colitis was induced in mice by continuous administration of 3.0% DSS in drinking water for 7 days (**Figure** **7**A). Subsequently, all mice were orally treated with HDCA and 12‐KCAC (50 mg k^−1^g) daily, based on optimal doses determined in previous studies. Compared to the Con group, oral administration of HDCA and 12‐KCAC significantly ameliorated DSS‐induced colitis as evidenced by a significantly reduced DAI, reduced weight loss, and ameliorated colon shortening (*p* < 0.05; Figure 7B–D). Histological analysis further revealed that colonic inflammatory cell infiltration, mucosal damage, and overall histological scores were attenuated in the HDCA‐ and 12‐KCAC‐treated group compared to the control group (Figure 7E). The gene expression levels of the pro‐inflammatory cytokines IL‐1β and IL‐6 in colonic tissue were significantly decreased (*p* < 0.001; Figure 7E), while the gene expression level of the anti‐inflammatory cytokine IL‐10 was significantly increased in the HDCA‐ and 12‐KCAC‐treated groups (*p* < 0.001; Figure 7E). Collectively, these results indicate that oral administration of HDCA and 12‐KCAC ameliorated clinical colitis symptoms and colon damage.

Consistent with the in vitro results, stimulation with HDCA and 12‐KCAC significantly inhibited the protein expression of Gucy1A transmembrane receptor in DSS‐induced colitis mice, significantly upregulated the expression of Gucy2C transmembrane receptor protein (*p* < 0.05; Figure 7H; Figure S10, Supporting Information), and increased the levels of Ca^2+^ and cGMP in colonic tissue by treatment with HDCA and 12‐KCAC (*p* < 0.001; Figure 7G,J). Stimulation with HDCA and 12‐KCAC also significantly upregulated the expression of PKG protein in colonic tissues (*p* < 0.001; Figure 7I,K), thereby inhibiting the downstream expression of mTORC1 key proteins, in particular the signaling of pT389‐S6K, pS473‐AKT, and p‐S6 (Figure 7L). These data further support the role of HDCA and 12‐KCAC in the prevention and treatment of DSS‐induced colitis.

## 3 Discussion

**Figure 8:**
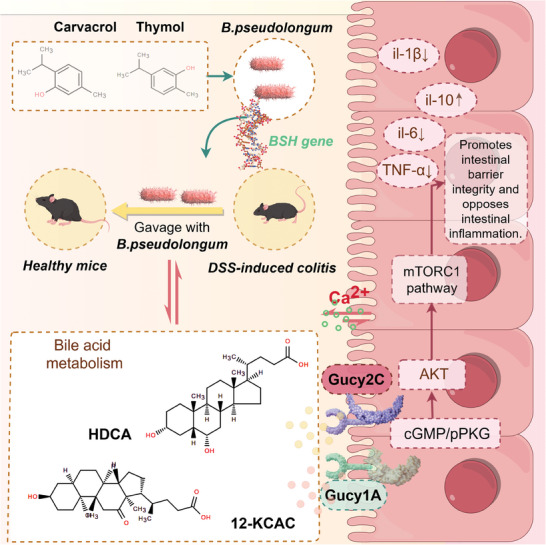
A model of the proposed mechanism by which CAT protects against colitis. This scheme illustrates that *Bifidobacterium pseudolongum*, as a targeted microbe of CAT, enhances the concentrations of HDCA and 12‐KCAC in colitis mice. These two key metabolites inhibit the expression of the colonic epithelial receptor protein Gucy1A, activate the expression of Gucy2C, elevate calcium ion and cGMP concentrations in the colon, and suppress the downstream mTORC1 pathway to counteract intestinal epithelial cell damage during colitis.

Phenolic compounds such as thymol and carvacrol, abundant in thyme and oregano plant extracts, have demonstrated significant antimicrobial, anti‐inflammatory, and antioxidant properties in animal models.^[^ ^21^ ^]^ Here we show that the anti‐inflammatory function of thymol and carvacrol in the gut is predominantly dependent on modulation by the gut microbiota, in particular the presence of *Bifidobacterium pseudolongum*. In vitro experiments demonstrated the ability of thymol and carvacrol to promote the growth of *B. pseudolongum*. However, in germ‐free animals lacking the targeted gut microbiota, thymol and carvacrol failed to exert their anti‐inflammatory effects. Notably, the *B. pseudolongum* genome encodes numerous genes involved in lipid metabolism, in particular the *BSH* gene, which facilitates the conversion of primary bile acids into secondary bile acids which has been linked to the alleviation of DSS‐induced colitis.^[^ ^22^ ^]^ Through extensive in vitro screening, we identified two critical bile acids, HDCA and 12‐KCAC, capable of inhibiting the expression of the transmembrane receptor protein Gucy1A in colonic epithelial cells. This inhibition subsequently increased the levels of Ca^2+^ and cGMP in colonic cells. The primary effector of cGMP‐PKGII is implicated in the inhibition of downstream mTORC1 signaling,^[^ ^23^ ^]^ which is critical for the alleviation of colitis. Knockdown of Gucy1A gene expression upregulated Gucy2C expression, thereby enhancing anti‐inflammatory effects. Abundant evidence supports the notion that the Gucy2C‐cGMP signaling axis counteracts intestinal epithelial damage and tumorigenesis^[^ ^24^ ^]^ underscoring the potential of targeting endogenous Gucy2C and Gucy1A transmembrane proteins in the later stages of colitis therapy. The results presented here highlight the pivotal role of phenolic compounds, particularly thymol and carvacrol, in alleviating intestinal inflammation, focusing on the intricate interplay between intestinal microbiota and host physiology. In particular, *B. pseudolongum* emerges as a critical mediator of the anti‐inflammatory effects of thymol and carvacrol, due to its genomic repertoire favoring bile acid metabolism. Furthermore, the elucidation of HDCA and 12‐KCAC as key bile acids underlines their potential therapeutic relevance in the treatment of colitis. Mechanistically, their ability to modulate Gucy1A expression and subsequent downstream signaling cascades sheds light on novel pathways for resolution of inflammation in the colon. Importantly, the interplay between the Gucy1A and Gucy2C transmembrane proteins emerges as a promising avenue for future therapeutic intervention in colitis (**Figure** **8**). Collectively, these findings highlight the intricate interplay between microbial metabolites, host signaling pathways, and inflammatory resolution in the gut, providing new avenues for targeted therapeutic intervention in colitis and related inflammatory diseases.

Through in vitro validation, this study confirmed that CAT supplementation promotes the proliferation of *B. pseudolongum*. Previous research has shown that intestinal *B. pseudolongum* can enhance the response to immunotherapy by producing the metabolite inosine. Immunotherapy induces a decrease in intestinal barrier function, increasing systemic transport of inosine and activated anti‐tumor T cells.^[^ ^25^ ^]^ In addition, *B. pseudolongum* significantly inhibits the formation of non‐alcoholic fatty liver disease‐associated hepatocellular carcinoma. The anti‐tumor effect is attributed to acetate, a metabolite produced by *B. pseudolongum*. Acetate binds to the G protein‐coupled receptor 43 of liver cells, activates GPR43, and inhibits the IL‐6/JAK1/STAT3 signaling pathway, thereby suppressing NAFLD‐HCC formation.^[^ ^26^ ^]^ Our study, which has been causally validated at the genomic level and in a mouse model of DSS‐induced colitis, confirms that *B. pseudolongum* promotes the production of secondary bile acids in the intestine, in particular the levels of HDCA and 12‐KCAC, to alleviate colitis. Taken together, these findings suggest that *B. pseudolongum* may serve as a potential probiotic for development and use. However, further probiotic safety assessment and human clinical trials are needed to systematically evaluate the safety of *B. pseudolongum* and its efficacy in the human gut, and thus provide crucial baseline data for the industrial application of this strain.

The Gucy1A gene encodes the alpha 1 subunit of soluble guanylate cyclase (sGC), which forms a heterodimeric enzyme with the encoded beta 1 subunit and serves as the primary receptor for nitric oxide (NO).^[^ ^27^ ^]^ The binding of NO to the haem iron of sGC induces the production of cGMP and subsequent activation of the cGMP‐ PKG pathway.^[^ ^12^ ^]^ Previous studies on the function of the Gucy1A gene have mainly focused on cardiovascular diseases,^[^ ^28^ ^]^ and its role in the regulation of intestinal inflammation remains largely unknown. Our study has shown that key bile acids, HDCA and 12‐KCAC, can inhibit the gene expression of intestinal epithelial Gucy1A, thereby modulating the occurrence of intestinal inflammation. This discovery is likely to prompt researchers to further investigate the broader functions of this gene in the gut. In contrast, extensive research has already elucidated the involvement of the Gucy2C gene in the regulation of intestinal inflammation and tumor development, revealing the dysregulation of the Gucy2C‐cGMP signaling axis in the pathogenesis of intestinal transport disorders, inflammatory bowel diseases, and colorectal cancer.^[^ ^29^, ^30^, ^31^ ^]^ According to our data, the Gucy1A protein is involved in regulating the expression of Gucy2C to modulate the concentrations of Ca^2+^ and cGMP in colon cells. However, the interaction between the protein structures of Gucy1A and Gucy2C remains elusive. Further research is warranted to systematically elucidate the detailed regulatory relationship between these two proteins, thereby providing a more theoretical basis for their potential application as key therapeutic targets for the treatment of colitis.

Despite the promising findings, our study has several limitations that warrant further investigation. The primary limitation lies in our incomplete understanding of the mechanistic pathway by which *B. pseudolongum* promotes the production of HDCA and 12‐KCAC. Although we demonstrated the involvement of *B. pseudolongum* in elevating these bile acids and linked this to anti‐inflammatory effects in colitis, the exact metabolic precursors and biosynthetic intermediates leading to HDCA and 12‐KCAC formation remain elusive. Current methodologies have not yet enabled us to identify the specific precursor molecules or elucidate the complete enzymatic processes involved. Addressing this gap is crucial, as understanding the biosynthetic pathway could provide targeted strategies for enhancing the therapeutic production of these metabolites. Future studies utilizing advanced metabolomic and genetic tracing approaches are necessary to fully delineate this mechanism.

## 4 Conclusion

In summary, utilizing the DSS‐induced colitis model, we have substantiated the effectiveness of CAT in mitigating colitis in murine subjects. Employing a pseudo‐germ‐free mouse model, we have established a direct association between CAT and its target, the bioactive gut bacterium *B. pseudolongum*, alongside its metabolites HDCA and 12‐KCAC. Notably, HDCA and 12‐KCAC ameliorate colitis by impeding the expression of Gucy1A in colonic epithelial cells, thereby elevating Ca^2+^ and cGMP levels, and activating cGMP‐PKGII to suppress the mTORC1 pathway, consequently mitigating colitis. This investigation enhances our comprehension of the molecular underpinnings of CAT in colitis treatment by modulating the gut microbiota and metabolites.

## 5 Experimental Section

### Animals

Seven‐week‐old female SPF C57BL/6J mice were obtained from the Northwest A&F University Animal Facility for experimental purposes. All experimental procedures involving animals were ethically approved by the Institutional Animal Care and Use Committee (IACUC) at Northwest A&F University to ensure compliance with established guidelines for the human treatment of laboratory animals (permit numbers: 2020‐06‐017). Prior to the start of the experiments, each mouse was individually housed in an independent ventilation cage for 1 week to acclimatize to the laboratory environment. During this acclimatization period, mice had ad libitum access to standard rodent chow and water (Table S1, Supporting Information). Initial body weights of the mice were recorded after the acclimatization period to provide baseline data for subsequent analyses. Mice were randomly assigned to experimental groups after the acclimatization period. Care was taken to ensure that there were no significant differences in initial body weight between groups, thereby minimizing potential confounding effects.

### DSS‐Induced Colitis Model Establishment

To induce chemical colitis in mice, a 2.5% (wt/vol) solution of DSS with a molecular weight range of 36 000–50 000 (MACKLIN, Shanghai, China) was prepared and provided ad libitum in the drinking water.^[^ ^32^ ^]^ At the onset of apparent diarrhea and significant weight loss compared to the control group, the DSS treatment was discontinued and the following experimental procedures were initiated.

### Oral CAT Treatment

A mixture of chemically synthesized carvacrol and thymol at a ratio of 1:1 was prepared for oral administration. Biosynthesized carvacrol and thymol in pure form were supplied by Novus International Inc (Missouri, USA). Mice were randomly divided into three groups: a negative control group receiving PBS every day for 14 days following 12 days of regular drinking water, a DSS‐only group receiving PBS similarly, and a CAT‐treated group receiving 40 µL kg^−1^ of the CAT mixture every day for 14 days following 12 days of DSS exposure.^[^ ^33^ ^]^ Body weights were recorded every 2 days, and diarrhea occurrence was monitored daily. At the conclusion of the experiment, mice were euthanized with CO_2_, and spleen weights were recorded. Serum, colonic contents, and colonic tissues were collected for further analyses, with serum stored at −20 °C and colonic contents/tissues preserved in liquid nitrogen.

### Pseudo‐Germ‐Free Mouse Model

To investigate the role of microbiota in colitis pathogenesis, female C57BL/6J mice were treated with a broad‐spectrum antibiotic mixture in their drinking water for 12 days. The antibiotic mixture consisted of 1 g L^−1^ ampicillin, 1 g L^−1^ streptomycin, 1 g L^−1^ gentamicin, and 0.5 g L^−1^ vancomycin (Macklin, Shanghai, China).^[^ ^34^ ^]^ Subsequently, the DSS‐induced colitis model was established using the same protocol, and the CAT mixture was administered according to the previously described procedure.

### Oral *Bifidobacterium pseudolongum* Treatment

To evaluate the therapeutic efficacy of *B. pseudolongum* RU224 in DSS‐induced colitis, mice were randomized into three groups: a normal control group receiving normal drinking water, a DSS+PBS group receiving DSS in the drinking water for 12 days followed by PBS administration every other day, and a DSS+BP group receiving 10^9^ CFU/animal/day of *B. pseudolongum* RU224 every other day after 12 days of DSS exposure. Body weights were recorded every 2 days and the incidence of diarrhea was monitored daily. At the end of the experiment, mice were euthanized and spleen weights were recorded. Serum, colonic contents, and colonic tissue were collected for further analysis.

### Oral Hyodeoxycholic Acid and 12‐Ketolithocholic Acid Treatment

To evaluate the therapeutic effects of HDCA and 12‐KCAC in DSS‐induced colitis, mice were randomly allocated into four groups: a normal control group receiving regular drinking water, a DSS+PBS group receiving 3% DSS in the drinking water for 7 days followed by PBS administration every other day, a DSS+HDCA group receiving 50 mg k^−1^g d^−1^ of HDCA, and a DSS+12‐KCAC group receiving 50 mg k^−1^g d^−1^ of 12‐KCAC. The doses of drug treatments were referred to previous studies.^[^ ^35^, ^36^ ^]^ HDCA was purchased from Shanghai Macklin Biochemical Technology Co., Ltd. (#H810982, Shanghai, China), and 12‐KCAC was obtained from Shanghai Yuan Ye Biotechnology Co., Ltd. (#S22145, Shanghai, China). Body weights were recorded every 2 days, and diarrhea occurrence was monitored daily. At the conclusion of the experiment, mice were euthanized, and spleen weights were recorded. Serum, colonic contents, and colonic tissues were collected for further analyses, with serum stored at −20 °C and colonic contents/tissues preserved in liquid nitrogen for subsequent analyses.

### Bacterial Culture Conditions

Fecal samples collected from mice after CAT treatment were dissolved in sterile PBS containing 30% glycerol and shaken vigorously. The samples were then serially diluted with sterile PBS to obtain dilutions of 10^−3^, 10^−5^, and 10^−7^. A volume of 20 µL from each dilution was then inoculated onto BSM agar plates supplemented with 0.05% (wt/vol) cysteine hydrochloride.^[^ ^37^ ^]^ The inoculated plates were then incubated anaerobically at 37 °C for 72 h. After 72 h of anaerobic incubation, colonies, typically 1 to 2 mm in diameter, were visible on the agar plates. Individual colonies were selected and inoculated onto fresh BHI agar plates to obtain pure cultures. Pure colonies obtained from the agar plates were inoculated into anaerobic tubes containing 10 mL of BSM broth medium. The composition of the medium per liter included 10 g sodium ascorbate, 10 g pancreatic digest of casein, 0.5 g cysteine hydrochloride, 10 g glucose, 5 g beef extract, 3 g potassium dihydrogen phosphate, 1 mL Tween‐80 and 5 g yeast extract. The tubes were then subjected to vacuum aspiration to remove oxygen and a CO_2_ atmosphere was established, followed by incubation on a horizontal shaker at 37 °C for 12–24 h. After incubation, when the bacterial culture became turbid, DNA extraction was performed using a microbial DNA extraction kit (#DP302‐02; Tiangen, Beijing, China) according to the manufacturer's instructions. PCR amplification of the extracted DNA was performed using universal bacterial primers 27F‐1492R. The PCR products were then sent to Shanghai Biotechnology Corporation (Shanghai, China) for sequencing. The sequencing data obtained were compared and analyzed with reference sequences available in the NCBI database to identify and confirm the strain *B. pseudolongum* RU224.

To further confirm whether *B. pseudolongum* RU224 promotes the production of 12‐KCAC and HDCA, in vitro fermentation was performed using BSM supplemented with either inactivated or fresh mouse feces. Briefly, BSM was then inoculated with either live or heat‐killed *B. pseudolongum* RU224 and incubated in an anaerobic workstation at 37 °C for 24 h. After incubation, the supernatants were collected, and the concentrations of 12‐KCAC and HDCA were determined using ELISA kits (kit numbers F0281‐MB and F0286‐MB; FANKEW, Shanghai, China).

### Cells and Stimulation Conditions

The HEK293T cell line was purchased from the National Science & Technology Infrastructure (Shanghai, China). HEK293T cells were cultured in Dulbecco's modified eagle medium (DMEM) supplemented with 10% fetal bovine serum at 37 °C in 5% CO_2_.^[^ ^38^ ^]^ Cells were maintained in a humidified incubator under standard cell culture conditions. To assess the anti‐inflammatory effects of different bile acids, cells were stimulated with lipopolysaccharide (LPS) at a concentration of 5 µg mL^−1^ for 24 h. After 24 h, the culture medium was replaced with LPS‐free medium, and the cells were treated with different concentrations (50, 75, and 100 µm) of hyodeoxycholic acid, 12‐ketolithocholic acid, 7‐ketolithocholic acid, α‐muricholic acid, β‐muricholic acid, apocholic acid, deoxycholic acid, 3‐epideoxycholic acid, ursodeoxycholic acid, and alocholic acid for 12, 24, or 36 h as indicated. Non‐specific control siRNA and Gucy1A3 and Gucy2C specific siRNA (GenePharma, Shanghai, China) were mixed with Lipofectamine 3000 in a 1:1 ratio. The mixture was diluted in serum‐free culture medium and incubated at room temperature for 30 min. After incubation, the mixture was added to six‐well plates and transfected for 24 h. After transfection, the cells were transferred to 12‐ or 24‐well plates and treated with LPS (5 µg mL^−1^) and 50 µm hyodeoxycholic acid or 12‐keto‐hocholic acid for a further 24 h. siRNA used: si‐GUCY1A3: TTCTGTTTGTCAGTCTCATATAA and si‐GUCY2C: CTCAGGAAAATTTCAAATGCACA.

### Determination of Cytokine Levels

Peritoneal cells were gently washed with cold PBS and then digested with trypsin. After centrifugation at 1000 ×* g* for 5 min, cells were collected. The collected cells were washed three times with cold PBS, resuspended in 150–200 µL PBS per 1 × 10^6^ cells, and subjected to repeated freeze‐thaw cycles to disrupt the cells. The cell lysates were then centrifuged at 1500 × *g* for 10 min, and the supernatants were collected for analysis. The concentrations of IL‐6, IL‐10, IL‐1β, and TNF‐α in mouse serum were determined using enzyme‐linked immunosorbent assay (ELISA) kits according to the manufacturer's instructions. The specific kit numbers were F2163‐B, F2176‐B, F2040‐B, and F2132‐B (FANKEW, Shanghai, China). Similarly, the concentrations of IL‐6, IL‐10, IL‐1β, and TNF‐α in cell culture supernatants were also measured using ELISA kits according to the manufacturer's instructions. The specific kit numbers were F0049‐A, F0065‐A, F0179‐A, and F0121‐A (FANKEW, Shanghai, China).

### Measurement of Cellular Ca^2+^ and cGMP Levels

Colon tissue collected was initially processed for homogenization. Specifically, the tissue was rinsed with pre‐cooled PBS (0.01 M, pH = 7.4) to remove residual blood. After weighing, the tissue was minced and homogenized with PBS containing protease inhibitors at a ratio of 1:9 (wt/vol). The homogenate was then sonicated to further disrupt the tissue structure. Finally, the homogenate was centrifuged at 5000 × *g* for 10 min, and the supernatant was collected for further analysis. The concentration of Ca^2+^ in the tissue homogenate was determined using a Ca^2+^ kit (C004‐2‐1, Jiancheng Bio, China) following the standard protocol provided by the manufacturer. Similarly, the concentration of cGMP was measured using a mouse cGMP ELISA kit (F2021‐B, FANKEW, Shanghai, China) according to the manufacturer's instructions. Additionally, the concentration of cGMP in the cell culture supernatant was also measured using a cGMP ELISA kit (F0197‐A, FANKEW, Shanghai, China) following the standard protocol provided.

### Histopathological Assessment

Colon tissue samples were fixed in 4% paraformaldehyde, followed by embedding in paraffin wax. Subsequently, tissue sections were cut and mounted onto glass slides. For histopathological analysis, tissue sections were stained with H&E to visualize cellular morphology and tissue architecture. H&E staining provides insights into tissue structure, inflammation, and any pathological changes present. To evaluate mucus production and goblet cell abundance, colon tissue sections were stained with PAS. PAS staining highlights the presence of acidic mucins in goblet cells, aiding in the assessment of mucosal integrity and secretory function. Stained tissue sections were observed under a light microscope (Carl Zeiss AG, Jena, Germany), and images were captured for further analysis. The images were examined to assess histological features, such as epithelial morphology, inflammatory cell infiltration, and goblet cell distribution. Goblet cells containing acidic mucins were identified and enumerated in PAS‐stained sections.

### 16S rRNA Gene Sequencing and Data Analysis

Colon contents were collected from the mouse model. Total DNA was extracted from colon contents using the E.Z.N.A. Stool DNA Kit (Omega Bio‐Tek, Norcross, GA, USA) following the manufacturer's instructions. The concentration and purity of DNA were determined using a Nanodrop 2000 UV–vis spectrophotometer (Thermo Scientific, Wilmington, USA). The quality of extracted DNA was assessed using 1% agarose gel electrophoresis. The V3–V4 region of DNA was amplified using the primers 338F (5′‐ACTCCTACGGGAGGCAGCAG‐3′) and 806R (5′‐GGACTACHVGGGTWTCTAAT‐3′) on a thermal cycler PCR system (Gene Amp 9700, ABI, USA). The amplified DNA was sequenced using the Illumina MiSeq platform (Illumina, San Diego, USA) following the standard protocols provided by Major Biobio‐Pharm Technology Co. Ltd (Shanghai, China).^[^ ^39^ ^]^ Sequence data in Illumina (fastq) format were imported into the QIIME2 platform (version 2020.2). Multiplexed sequences were demultiplexed using FLASH software (version 1.2.11)^[^ ^40^ ^]^ and then merged into paired‐end reads using FASTP software.^[^ ^41^ ^]^ The DADA2 plugin in QIIME2 was used for filtering, denoising, merging, and removing chimeras from the sequences. Taxonomic classification of amplicon sequence variants (ASVs) was performed using the naive Bayes consensus taxonomy classifier trained on the SILVA 138/16s_bacteria database in QIIME2.^[^ ^42^ ^]^ Alpha diversity analysis of microbial communities was conducted using the Kruskal–Wallis rank‐sum test followed by false discovery rate (FDR) correction. Beta diversity analysis was performed using unweighted UniFrac distance metric, and inter‐group differences were assessed using ANOSIM with 999 permutations. Differential abundance analysis at different taxonomic levels was conducted using the Kruskal–Wallis H test with FDR correction, and post‐hoc tests were performed using the Tukey–Kramer method with a threshold of 0.95. Spearman correlation network analysis was conducted using python‐2.7 (stat) software, with an absolute correlation coefficient threshold set to 0.5.

### RNA Extraction and RNA‐Sequencing

Total RNA was extracted from mouse colonic epithelial tissue using the TRIzol (Invitrogen) method. The concentration and purity of the extracted RNA were measured using a Nanodrop 2000 spectrophotometer, while RNA integrity was assessed by agarose gel electrophoresis. Additionally, the RNA integrity number (RIN) was determined using an Agilent 2100 Bioanalyzer, with all samples showing clear bands, free of contaminants, and RIN values above 8.5, meeting the criteria for subsequent library construction. Libraries were constructed following the standard protocol of Shanghai Majorbio Bio‐pharm Technology Co., Ltd. The constructed libraries were subjected to high‐throughput sequencing using the Illumina NovaSeq 6000 platform, with sequencing reads of 150 bp in length. After sequencing, SEQPREP software was utilized to trim adapter sequences from the 3′ and 5′ ends of reads. Sickle (Version 1.33) software was then employed to filter out reads with lengths less than 20 bp, average base quality scores below 20, and reads containing N bases, retaining high‐quality reads. Subsequently, TopHat software^[^ ^43^ ^]^ was used to align the clean data to the mouse reference genome, yielding mapped data. The mapped data were utilized for transcriptome assembly and expression quantification. RSEM software^[^ ^44^ ^]^ was employed for accurate quantification of gene and transcript expression levels. DESeq2 (Version 1.24.0) software was used to identify differentially expressed genes (DEGs) between experimental conditions.^[^ ^45^ ^]^ The criteria for DEG selection were set as *p*‐adjust <0.05 and |log2FC| ≥ 1. GOATOOLS and KOBAS software were employed to perform functional enrichment analysis of the identified DEGs.^[^ ^46^ ^]^ To rigorously control for false positives during the calculation process, Bonferroni correction was applied for multiple testing, with a corrected *p*‐value ≤0.05 considered as significantly enriched pathways.

### Quantitative Reverse Transcriptase PCR

The total RNA was then reverse‐transcribed using the RevertAid First Strand cDNA Synthesis Kit (Thermo Fisher Scientific, USA) to obtain cDNA. qRT‐PCR was performed using ChamQ Universal SYBR qPCR Master Mix (Vazyme, China) in the Light Cycler®96 Real‐Time PCR System (Roche, USA). All primers used in this study were designed and preliminarily verified using Oligo 7 software and Primer‐BLAST (https://www.ncbi.nlm.nih.gov/tools/primer‐blast/). The primers were synthesized by Zhongke Yutong (Xi Bostan, China) company (Table S2, Supporting Information). The comparative cycle method (2 − ΔΔCt) was used to determine relative mRNA expression. The total population of bacteria and *B. pseudolongum* RU224 was enumerated by real‐time PCR according to the method of the previous study.^[^ ^47^ ^]^ The primers for total bacteria were as follows: bacF (5′‐ AGAGTTTGATCCTGGCTCAG‐3′) and bacR (5′‐ GGTTACCTTGTTACGACTT‐3′). The primers for *B. pseudolongum* RU224 were as follows: bacF (5′‐ ACAATATAGCACAGGGGTGGA‐3′) and bacR (5′‐ AGACATCGTGGAAACTGGGG‐3′).

### Colonic Content Metabolite Measurements Using LC‐MS/MS

Colon content samples were thawed on ice and 50 mg (±1 mg) of each sample was homogenized with 500 µL of ice‐cold methanol/water (70%, vol/vol). The samples underwent vortexing for 3 min, sonication for 10 min in an ice water bath, and vortexing again for 1 min. Subsequently, the samples were centrifuged (12 000 rpm) at 4 °C for 10 min, and 250 µL of the supernatant was collected and centrifuged again (12 000 rpm) at 4 °C for 5 min. Finally, 150 µL of the supernatant was taken for onboard analysis. The sample extracts were analyzed using an UHPLC‐ESI‐MS/MS system (UHPLC, ExionLC AD; ESI; QTRAP System). The UHPLC conditions were set as follows^[^ ^48^ ^]^: column, Waters ACQUITY UPLC HSS T3 C18; column temperature, 40 °C; flow rate, 0.4 mL mi^−1^n; injection volume, 2 µL; solvent system: solvent A (water with 0.1% formic acid) and solvent B (acetonitrile with 0.1% formic acid). The gradient program was as follows: 95:5 vol/vol at 0 min, 10:90 vol/vol at 10.0 min, 10:90 vol/vol at 11.0 min, 95:5 vol/V at 11.1 min, and 95:5 vol/vol at 14.0 min. MS/MS scans were acquired in both positive and negative ion modes. LC‐MS/MS data were processed using the Analyst 1.6.3 software package.^[^ ^49^ ^]^ Metabolites were identified based on a self‐built MWDB (METWARE database) and annotated using the KEGG compound database. Significantly regulated metabolites were determined by VIP >1 and absolute log2FC ≥1.^[^ ^50^ ^]^ The identified metabolites were mapped to the KEGG pathway database, and significantly enriched pathways were identified using the hypergeometric test.

### Protein Extraction and Peptide Fractionation

Frozen colonic tissue samples were cut into 1 cm pieces using a surgical blade and transferred to 1.5 mL tubes. The tissue samples were lysed with 500 µL of digestion buffer (containing 1 mm PMSF) and homogenized on ice. Further disruption was achieved by sonication. Subsequently, the samples were centrifuged at 15 000 × *g* at 4 °C for 15 min to remove insoluble particles and debris. Protein concentration was determined using the Bicinchoninic Acid method, and the samples were stored at −80 °C. For protein digestion, 10 µg of protein from each sample was digested with 3 µL of sequencing‐grade trypsin (1 µg µL^−1^) in 100 µL of 300 mm TEAB buffer at 37 °C for 12 h. Following digestion, 40 µL of each sample was transferred to a new tube for TMT labeling. TMT labeling reagents were added to each sample, followed by incubation at room temperature for 1 h. The reaction was quenched by adding 8 µL of 5% hydroxylamine and incubating for 15 min. The TMT‐labeled peptides were separated using an Agilent 1100 HPLC system and analyzed on a SCIEX TripleTOF 5600 mass spectrometer equipped with a NanoSpray III source.^[^ ^51^ ^]^ Peptides were fractionated using a reverse‐phase C18 column with a high‐pH mobile phase gradient. The LC gradient program consisted of various proportions of solvent A (2% acetonitrile with ammonium hydroxide, pH 10) and solvent B (80% acetonitrile with ammonium hydroxide, pH 10). UV detection was performed at 214 nm, and the flow rate was 200 µL mi^−1^n. The total LC‐MS/MS analysis time was 60 min. For the second‐dimension analysis, peptides were dissolved in the mass spectrometry loading buffer and separated using a C18 chromatographic column (75 µm × 25 cm, Thermo, USA) on an Easy‐nLC 1200 system coupled with a Q Exactive mass spectrometer.^[^ ^52^ ^]^ The peptides were eluted using a gradient of solvent A (2% acetonitrile with 0.1% formic acid) and solvent B (80% acetonitrile with 0.1% formic acid) over 120 min. MS and MS/MS data were acquired with automatic switching between full scan and top20 data‐dependent acquisition modes, with mass resolutions of 70 and 35 K, respectively. Full scans were performed in the m/z range of 350–1300, and the top 20 precursor ions were selected for fragmentation. Dynamic exclusion was set to 18s.

### Proteomic Data Analysis

The raw files obtained from mass spectrometry were analyzed using Proteome Discoverer 2.2 (Thermo Fisher Scientific, MA, USA) for protein identification and quantification. The peptide identification FDR during database searching was set to FDR ≤ 0.01. Proteins were considered identified if they contained at least one unique peptide.^[^ ^53^ ^]^ A total of 8760 proteins were detected in this study. Using the T.test function in the R programming language, significance *p*‐values for differences between samples were calculated. Additionally, fold changes (FC) between groups were computed. Criteria for selecting significantly differentially expressed proteins were as follows: proteins with *p* < 0.05 and FC > 1.2 were considered upregulated, while proteins with *p* < 0.05 and FC < 0.83 were considered downregulated. Differential proteins were functionally annotated and subjected to biological function analysis using DIAMOND (v0.8.37.99), a tool for fast and sensitive protein alignment. This analysis provided insights into the biological functions and pathways associated with the identified differentially expressed proteins.

### Bacterial Whole Genome Sequencing and Analysis

The whole‐genome data of *B. pseudolongum* RU224 strain were obtained from NCBI, with Biosample Accession: PRJNA391001. The bacterial genome was assembled using the Unicycler assembly software for hybrid assembly of third‐generation sequences. Sequence correction during assembly was performed using Pilonjin software. Gene prediction in the assembled genome was conducted using Glimmer^[^ ^54^ ^]^ and GeneMarkS software.^[^ ^55^ ^]^ Functional genes, including nucleotide and amino acid sequences, were predicted from the assembled genome using Glimmer and GeneMarkS software. These predicted genes were used for subsequent functional analysis. tRNA genes in the genome were predicted using tRNAscan‐SE v2.0 software,^[^ ^56^ ^]^ while rRNA genes were predicted using Barrnap software. Predicted coding genes were annotated by similarity comparison with functional databases using protein sequences. Annotations obtained included NR, Swiss‐Prot, Pfam, COG, GO, and KEGG information, with a threshold set at E‐value ≤ 1E‐5. A circular genome map of *B. pseudolongum* RU224 strain was generated using CGView software for visualization and exploration. Carbohydrate‐active enzymes were analyzed and annotated using Diamond^[^ ^57^ ^]^ and hmmscan software,^[^ ^58^ ^]^ with an E‐value cutoff set at ≤ 1E‐5.

### Western Blotting

Colon tissues were weighed and homogenized in cell lysis buffer (Beyotime, China). The homogenate was then centrifuged at 15 000 × *g* and 4 °C for 15 min to obtain the supernatant. Omni‐Easy™ instant BCA protein assay kits (Epizyme, China) were used to quantify the protein concentration following the manufacturer's instructions. The proteins were separated using 10% SDS polyacrylamide gel and then transferred onto PVDF membranes. The membranes were blocked with 5% skimmed milk for 1 h, then immunoblotted with primary antibodies against pT389‐S6K (1:1000, #9234, CST, USA), p‐S6 (1:1000, #4858, CST, USA), S6K (1:1000, #9202, CST, USA), S6 (1:1000, #2217, CST, USA), p‐ERK (1:1000, #4370, CST, USA), ERK (1:1000, #4695, CST, USA), pS473‐AKT (1:1000, #4060, CST, USA), AKT (1:1000, #9272, CST, USA), Gucy1A3 (1:1000, #12605‐1‐AP, Proteintech, China), Gucy2C (1:1000, #C27477, SAB, USA), PKG2(1:100, 55138‐1‐AP, Proteintech, China)and Actin (1:1000, #20536‐1‐AP, Proteintech, USA) at 4 °C overnight. The membranes were washed three times with 1x TBST (T1081, Solarbio, China) for 5 min each time and then incubated with secondary antibodies labeled with HRP at room temperature for 1 h. The bands were visualized using Omni‐ ECL™pico light chemiluminescence kits (Epizyme, China). Actin was used as the reference protein. All blots or gels were derived from the same experiment and were processed in parallel.

### Immunofluorescence Staining

The tissue sections were subjected to two incubation steps in xylene, each lasting 20 min, followed by two incubation steps in absolute ethanol, each lasting 5 min, and a 5‐min incubation in 75% ethanol. Subsequently, the sections were washed with distilled water and subjected to antigen retrieval in a sodium citrate solution (2.94 g sodium citrate tribasic dihydrate in 1 L distilled water with 500 µL Tween‐20, pH 6) at 95 °C for 20 min After natural cooling, the slides were blocked with 5% bovine serum albumin (BSA) at room temperature for 1 h. Following removal of the blocking solution, the primary antibodies against Gucy1A3 (1:200, #12605‐1‐AP, Proteintech, China) and Gucy2C (1:200, #C27477, SAB, USA) were applied onto the slides and incubated overnight at 4 °C. After washing the slides with phosphate‐buffered saline (PBS), the corresponding secondary antibodies were applied and incubated at room temperature for 2 h. Subsequently, the slides were stained with DAPI for nuclear staining and incubated at room temperature for 10 min. The sections were then observed and images were captured using a fluorescence microscope (PannoramicMIDI 3DHISTEC, Danjier, Shandong, China) with excitation wavelengths of 330–380 nm for DAPI (emission at 420 nm, emitting blue light) and 465–495 nm for FITC (emission at 515–555 nm, emitting green light). Cells were cultured in a 24‐well cell culture plate until reaching confluence. After removing the culture medium, the cells were washed once with PBS and fixed with pre‐cooled 4 °C paraformaldehyde for 10 min. Subsequently, the cells were rinsed three times with PBS containing 0.5% Triton X‐100. The cells were then blocked with donkey serum diluted in PBS (3% donkey serum in PBS) for 30 min. Primary antibodies against Gucy1A3 (1:200, #12605‐1‐AP, Proteintech, China) and Gucy2C (1:200, #C27477, SAB, USA) were applied and incubated at 37 °C for 1 h, followed by incubation with corresponding secondary antibodies and DAPI (1:200, #C0060, Solarbio, China) at room temperature for 2 h. Finally, the cells were observed using an inverted fluorescence microscope (OLYMPUS, IX73, TUCSEN, Fujian, China).

### Statistical Analyses

GraphPad Prism (v 8.03, GraphPad, USA) was used to generate histograms. Statistical analysis was performed by the two‐tailed unpaired Student's *t*‐test or one‐way ANOVA followed by Tukey's multiple comparison test using SPSS software (version 20.0, SPSS, Chicago, IL, USA). Differences with a *p*‐value < 0.05 were considered significant. The Kruskal–Wallis H test was used to identify significant differences in the relative abundance at different taxonomic levels between groups. Linear discriminant analysis effect size (LEfSe) analysis was performed to identify the microbiome with higher relative abundance in the two groups. The non‐parametric factorial Kruskal–Wallis's rank sum test was used, followed by linear discriminant analysis (LDA) to evaluate the impact of each taxon abundance on the differential effect. A significant increase in microbiota abundance was defined as an LDA score (log10) greater than 3.0. Metabolites with VIP ≥ 1 and *p‐*value < 0.05 were generally considered to be significantly different.

## Conflict of Interest

The authors declare no conflict of interest

## Author Contributions

K.Z., Y.X., and Y.Z. contributed equally to this work. K.Z., Y.X., T.Z., X. C, and Y.T.Y. performed mouse experiments. K.Z. and Y.X. performed statistical analyses. Y.X., X.Z., and M.J. performed cell experiments. Y.C., Y.W., Y.X.Y., Z.L., X.W., and L.D. contributed to the conception, planning, and execution of experiments and analyses. F.Y. provided carvacrol and thymol samples. Y.X. and K.Z. wrote the manuscript. Y.C., D.B., X.W., L.D., and X.W. revised the paper. All authors read, edited, and approved the final manuscript. K.Z. served as the lead contact for the work.

## Supporting information



Supporting Information

## Data Availability

The metagenomic sequencing and RNA‐seq data are available from the national center for biotechnology information (NCBI) under accessions PRJNA1090619, PRJNA1091445, PRJNA1091786 and PRJNA1091939. The proteome resource is available from iProX (https://www.iprox.cn/page/home.html) under accessions IPX0008475000.
