# Supplementary material for: Bifidobacterium pseudolongum‐Derived Bile Acid from Dietary Carvacrol and Thymol Supplementation Attenuates Colitis via cGMP‐PKG‐mTORC1 Pathway
Source: Adv Sci (Weinh). 2024 Sep 23;11(43):2406917. doi: 10.1002/advs.202406917 (PMC11578315; doi:10.1002/advs.202406917)
Supplement: Supplementary file 1 — Supporting Information [file ADVS-11-2406917-s001.docx]

Supplementary Materials for

***Bifidobacterium pseudolongum*-derived bile acid from dietary carvacrol and thymol supplementation attenuates colitis via cGMP-PKG-mTORC1 pathway**

Ke Zhang *et al.*

*Corresponding author. Email: yangyuxin2002@126.com

**This PDF file includes:**

Figs. S1 to S10

Tables S1 to S2


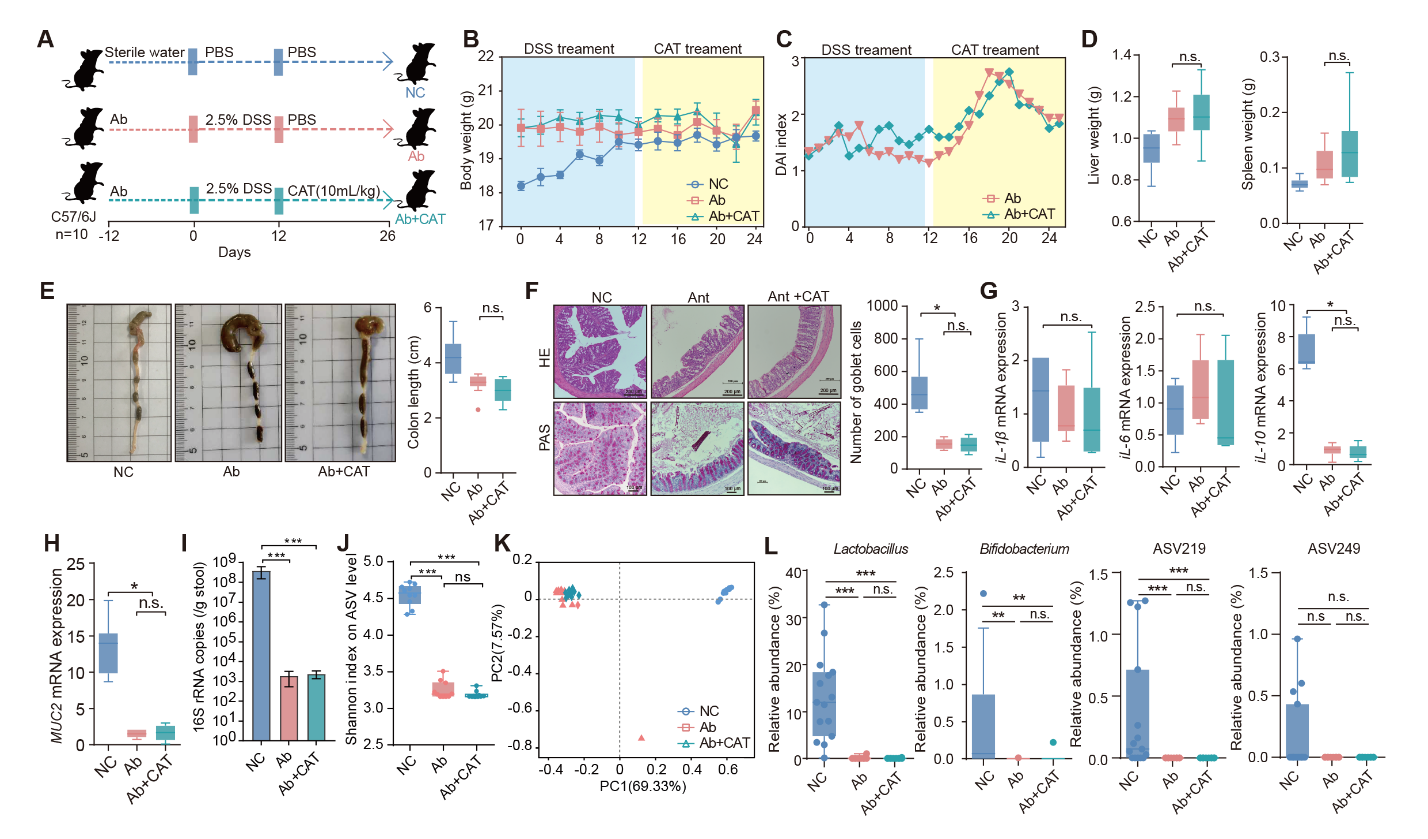


**Fig. S1.** **Validation of CAT-mediated gut microbial attenuation of DSS-induced colitis in a pseudo-sterile mouse model. (A)** Mice (male, n = 10 per group) were treated with antibiotic complex for 12 consecutive days and with 2.5% DSS in the presence or absence of CAT (40 μL/kg/d) for 14 consecutive days. **(B)** The body weight changes during the experiments were monitored. Mean ± SEM from three independent experiments. **(C)** The DAI index changes during the experiments were monitored. **(D)** Evaluation of spleen and liver weight. Statistical significance was determined using one-way ANOVA, followed by Tukey's test. **p* < 0.05. **(E)** Colon tissues were isolated on the last day of the experiment. A representative photograph of colon tissue from each group is provided, and the colon length was recorded. **(F)** The histological analysis of mouse colon tissue was performed by H&E, and alcian blue staining. Scale bar = 200 μm. Number of goblet cells of colon tissue were evaluated (n=5). **(G)** The mRNA level of *IL-1β*, *IL-6*, and *IL-10* in the mouse colon was assessed by qPCR analysis (n=10). **p* < 0.05, ***p* < 0.01, ****p* < 0.001. Statistical significance was determined using one-way ANOVA, followed by Tukey test. **(H)** The mRNA level of *MUC2* in the mouse colon was assessed by qPCR analysis (n=10). **(I)** Bacterial density as measured using quantitative PCR of 16S rRNA. **(J)** α-diversity upon oral therapy represented by the Shannon index (n = 10 per group). Statistical significance was determined using one-way ANOVA, followed by Tukey test. **p* < 0.05, ***p* < 0.01, ****p*< 0.001. **(K)** PCoA plot based on ASV abundances (n = 10 per group). The colors of the symbols indicate different treatment group, respectively. An analysis of similarity (ANOSIM) was used to assess the dissimilarity of Bray–Curtis. **(L)** The relative abundance of key microbes of *Lactobacillus*, *Bifidobacterium*, ASV219 and ASV249. Statistical significance was determined using a pairwise Wilcoxon test and two-tailed Fisher’s test with FDR correction (n = 10 per group). **p* < 0.05, ***p* < 0.01, ****p*< 0.001.


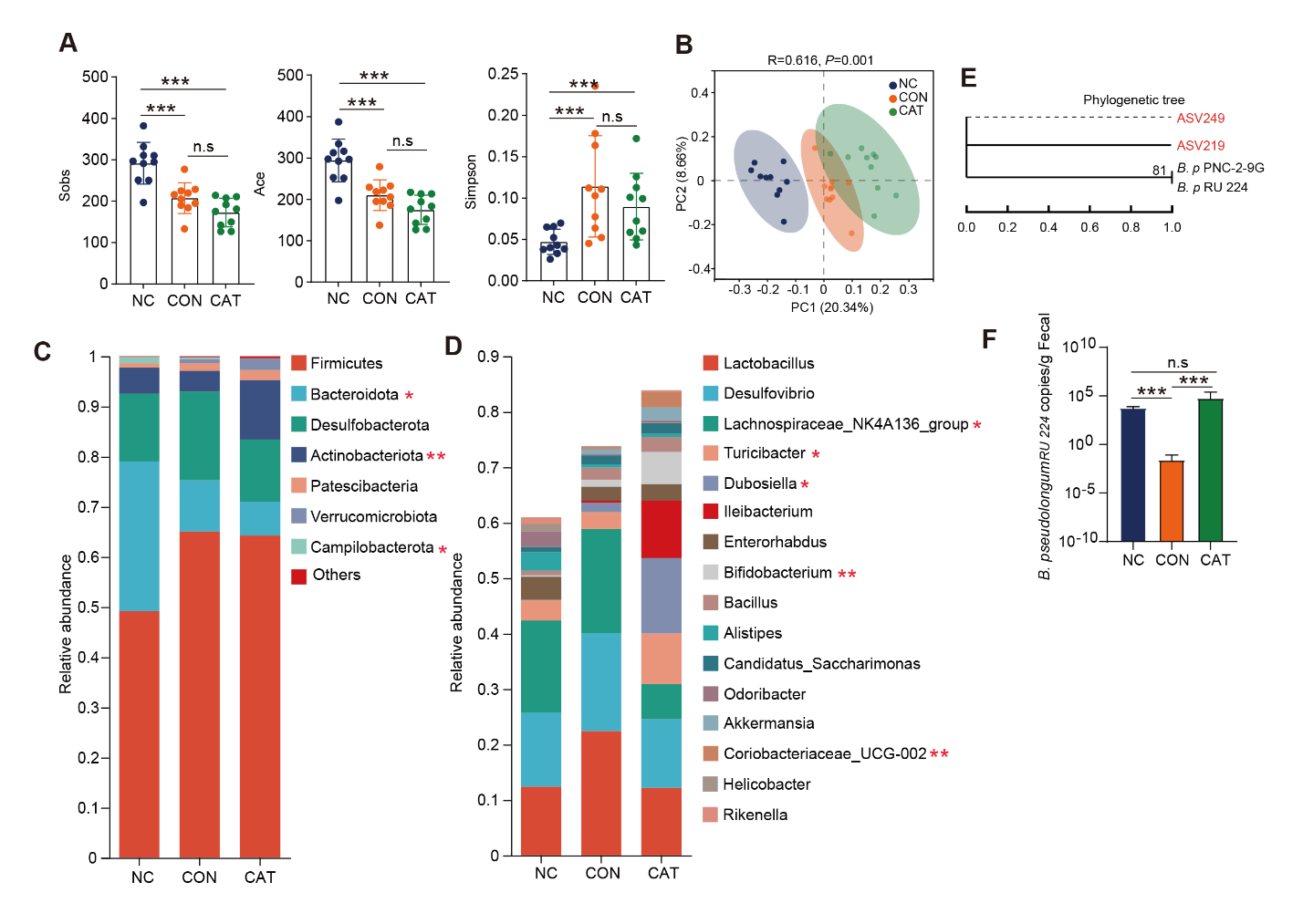
**Fig. S2. CAT alleviated DSS‑induced colitis in a gut microbiota–dependent manner.** **(A)** α-diversity upon oral therapy represented by the Shannon, Ace and Simpson index (n = 10 per group). Statistical significance was determined using one-way ANOVA, followed by Tukey test. **p* < 0.05, ***p* < 0.01, ****p*< 0.001. **(B)** PCoA plot based on ASV abundances (n = 10 per group). The colors of the symbols indicate different treatment group, respectively. An analysis of similarity (ANOSIM) was used to assess the dissimilarity of unweighted UniFrac distances. **(C)** The relative abundance of key microbes in phyla level. Statistical significance was determined using a pairwise Wilcoxon test and two-tailed Fisher’s test with FDR correction (n = 10 per group). **p* < 0.05, ***p* < 0.01, ****p*< 0.001. **(D)** The relative abundance of key microbes in genus level. Statistical significance was determined using a pairwise Wilcoxon test and two-tailed Fisher’s test with FDR correction (n = 10 per group). **p* < 0.05, ***p* < 0.01, ****p*< 0.001. **(E)** The phylogenetic tree of ASV249, ASV219, *Bifidobacterium pseudolongum* PNG-2-9G*,* and *Bifidobacterium pseudolongum* Ru 224. **(F)** *Bifidobacterium pseudolongum* Ru 224 density as measured using quantitative PCR. Statistical significance was determined using one-way ANOVA, followed by Tukey test. **p* < 0.05, ***p* < 0.01, ****p*< 0.001.


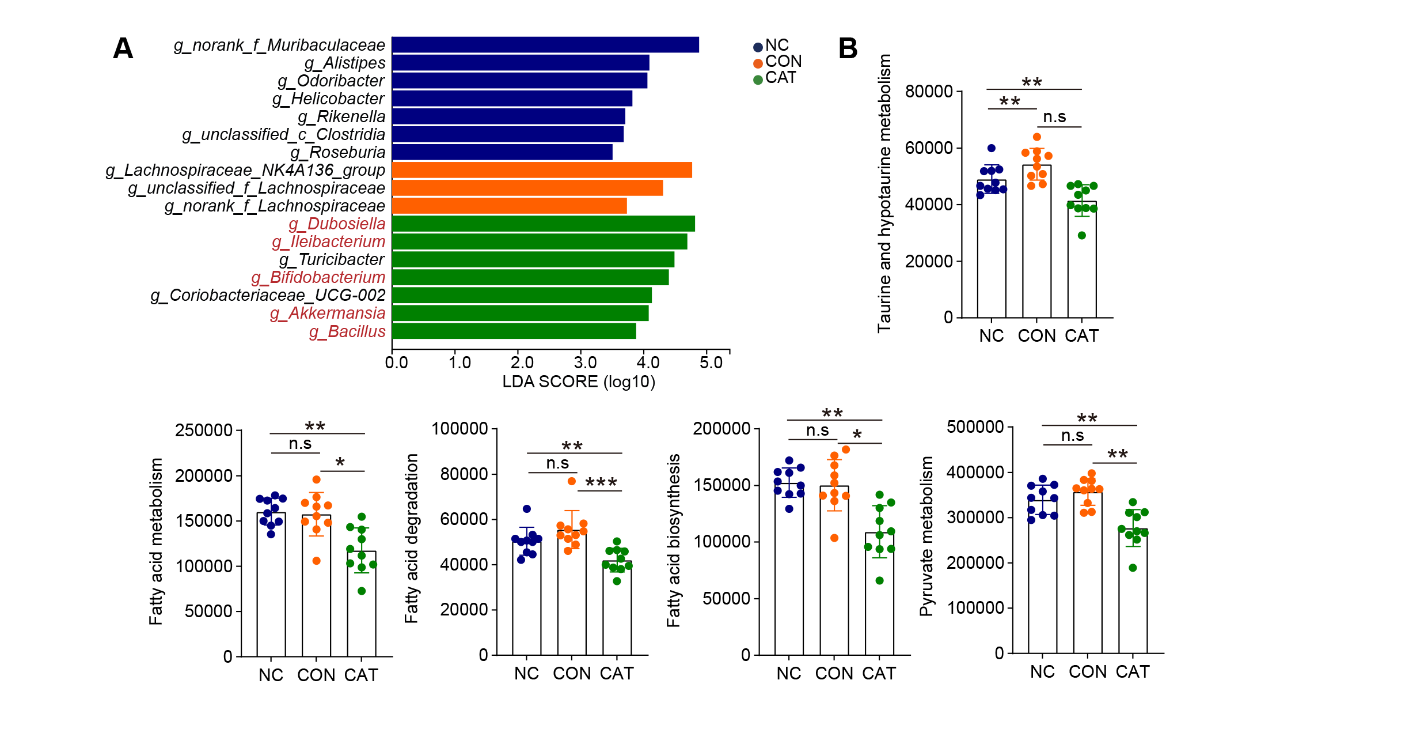


Fig. S3. CAT alters gut microbial composition and function in mice with DSS-induced colitis. (A) Differential abundance analysis using LEfSe identified bacterial taxa with differential abundance in response to oral CAT treatment in DSS-treated mice, LDA>3. (B) Functional prediction analysis of gut microbiota based PICRUSt. Statistical significance was determined using a pairwise Wilcoxon test (n = 10 per group). **p* < 0.05, ***p* < 0.01, ****p*< 0.001.


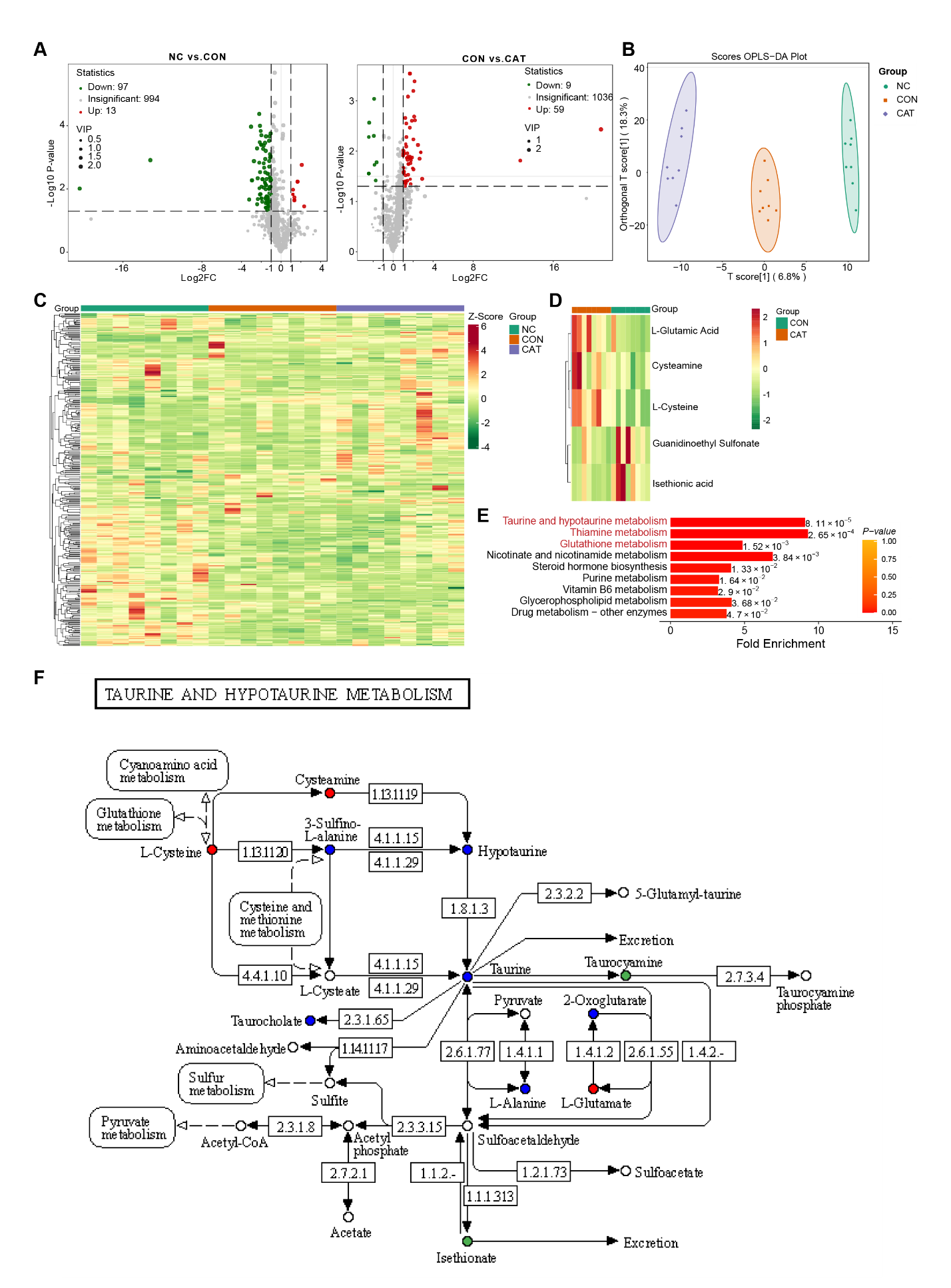


**Fig. S4. CAT-mediated alteration of gut microbiota composition modulates the colonic metabolome to alleviate DSS-induced colitis. (A)** Volcano plot illustrating the screening of differential metabolites between NC vs. Con and Con vs. CAT groups (n = 10 per group). Statistical significance was established with an absolute value of Log2FC > 1, denoted by **p* < 0.05. **(B)** OPLS-DA plot depicting the distinction in metabolic profiles among the three groups. **(C)** Heatmap representing differentially expressed metabolites among the groups, with significantly regulated metabolites defined by VIP > 1 and an absolute log2 fold change ≥ 1. **(D)** Differential distribution of key metabolites, L-Glutamic Acid, Cysteamine, L-Cysteine, guanidinoethyl sulfonate, and isethionic acid, between the CAT and Con groups. **(E)** Metabolite set enrichment analysis (MSEA) pinpointing divergent metabolic pathways between the two groups. **(F)** Illustration of altered metabolites within the "Taurine and hypotaurine metabolism" pathway, where red signifies significant upregulation in the CAT group compared to controls, and green denotes significant downregulation.


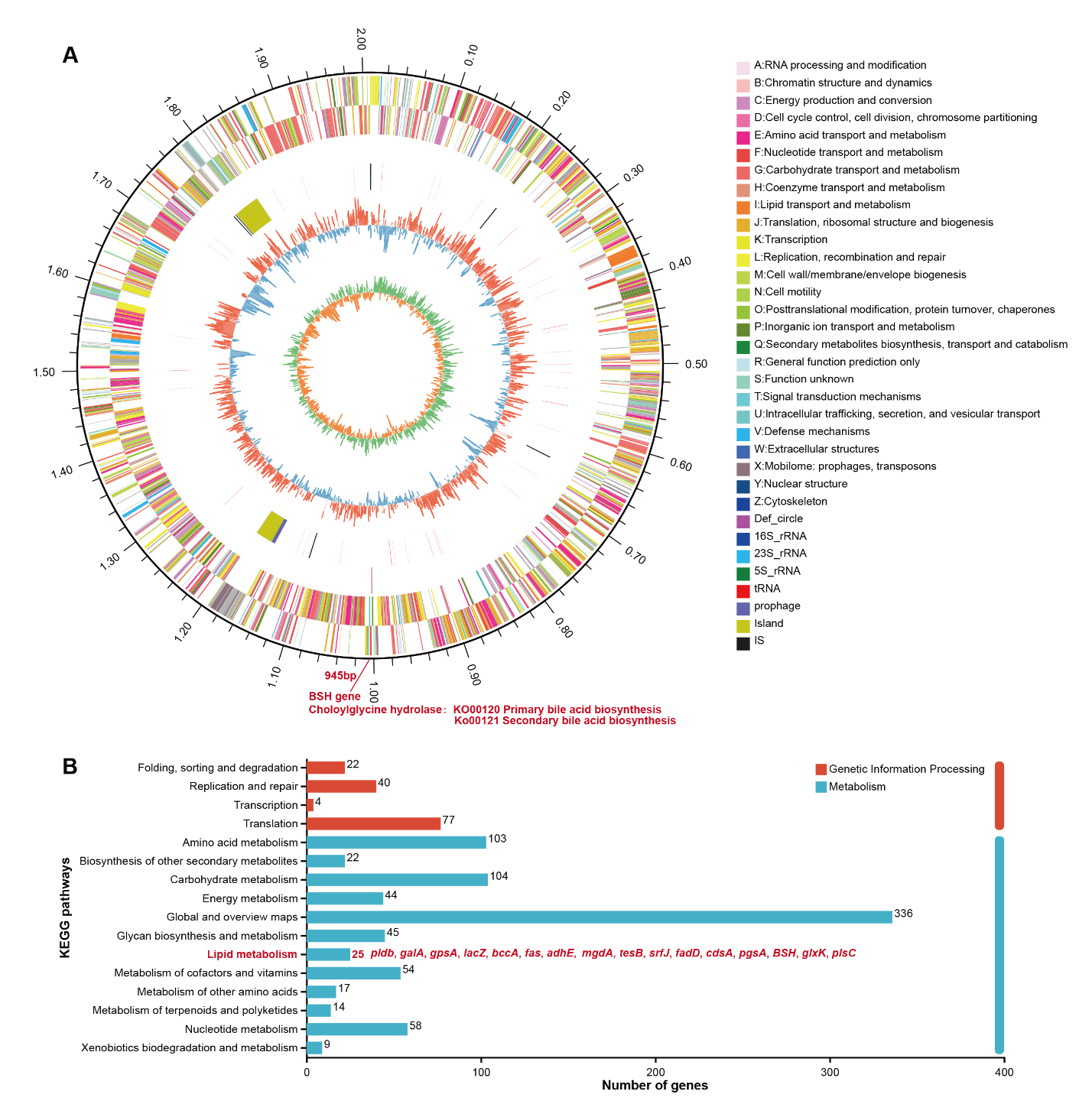


**Fig. S5. Functional annotation and genomic analysis of *Bifidobacterium pseudolongum* Ru 224. (A) Circular genomic map of *Bifidobacterium pseudolongum* Ru 224.** The outermost circle denotes the genome size. The second and third circles represent the coding sequences (CDS) on the positive and negative strands, respectively, color-coded according to the functional classification of Clusters of Orthologous Groups (COGs). The fourth circle indicates the locations of ribosomal RNA (rRNA) and transfer RNA (tRNA) genes. The fifth circle reflects the GC content: regions in red extending outward show GC content higher than the genome average, with taller peaks indicating a greater deviation; conversely, inward blue regions denote areas with GC content lower than the average, and taller peaks signify a more substantial difference. The innermost circle displays the GC skew, indicative of the bias between the frequencies of G and C nucleotides across the genome. **(B)** KEGG pathway annotation classification statistics. This bar chart illustrates the distribution of gene annotations into KEGG pathways. The vertical axis represents the second-level hierarchy of KEGG pathway classifications, while the horizontal axis shows the count of genes annotated under each category. Distinct bar colors denote the first-level classification of KEGG pathways, providing a visual summary of the functional diversity within the *B. pseudolongum* Ru 224 genome.


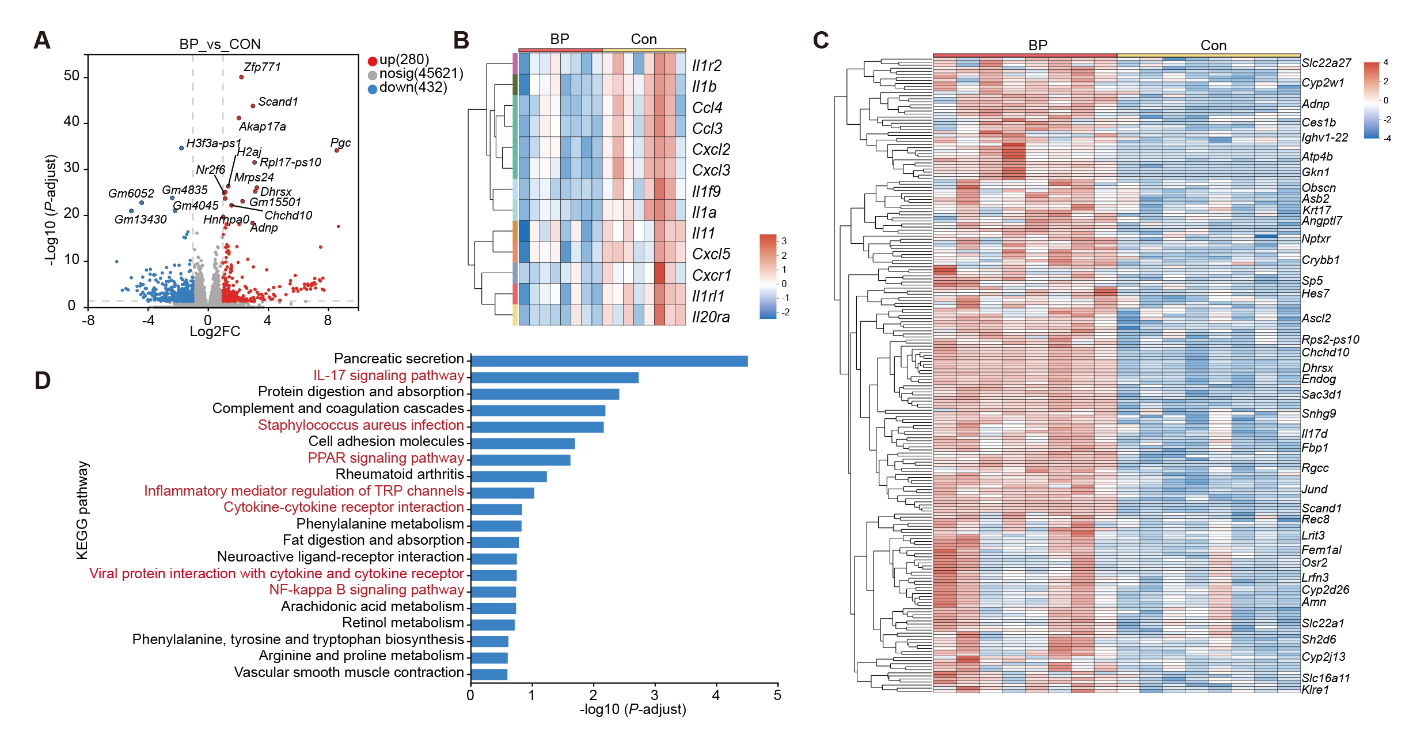


**Fig. S6. Transcriptomic analysis of colonic tissue revealing the molecular basis of *B. pseudolongum* Ru 224 in alleviating DSS-induced colitis. (A)** Volcano plot for differential gene expression analysis between BP and Con groups. In this plot, red dots symbolize genes significantly upregulated in the control (Con) group, whereas green dots represent those downregulated, based on the results from RNA-Seq. **(B)** Expression profile of genes associated with inflammatory responses. This section showcases the differential expression pattern of genes directly implicated in the initiation or regulation of inflammatory processes. **(C)** Expression profile of significantly upregulated differentially expressed genes (DEGs) in the BP group. It highlights the genes showing remarkable elevation in expression levels within the group administered *B. pseudolongum* Ru 224, suggesting their potential involvement in the protective mechanism against colitis. **(D)** KEGG enrichment analysis comparing BP to Con groups. This analysis identifies the enriched KEGG pathways among the DEGs, offering insights into the biological pathways and functions that are predominantly affected or regulated by *B. pseudolongum* Ru 224 intervention, further elucidating its mechanism in mitigating DSS-induced colitis.


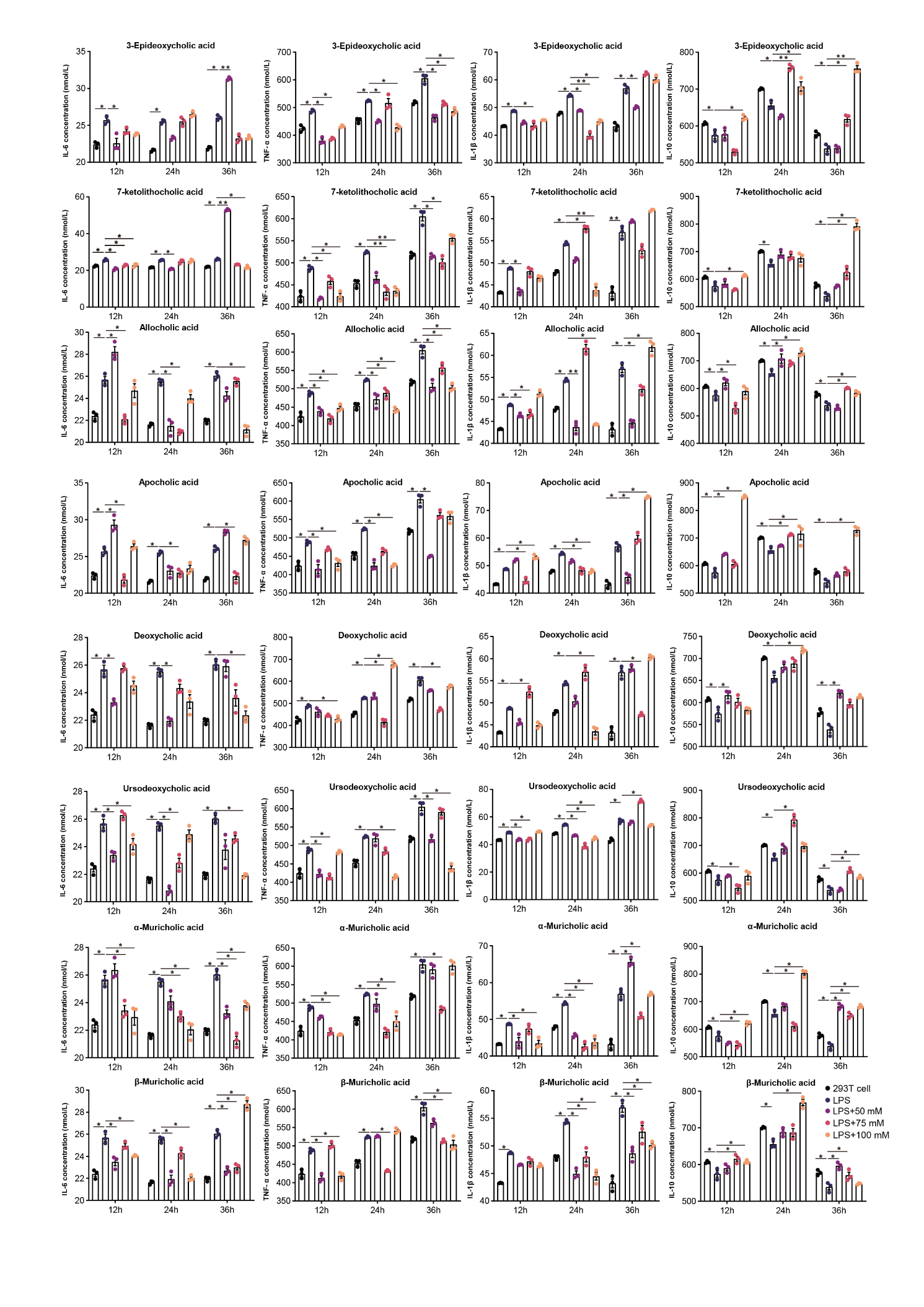


**Fig. S7. Changes in the concentrations of pro-inflammatory and anti-inflammatory cytokines in LPS-induced 293T inflammatory cell model upon treatment with key bile acid.** Mainly including 3-Epideoxycholic acid, 7-ketolithocholic acid, Allocholic acid, Apocholic acid, Deoxycholic acid, Ursodeoxycholic acid, α-Muricholic acid, andβ-Muricholic acid**.** Statistical significance was determined using one-way ANOVA, followed by Tukey test. **p* < 0.05, ***p* < 0.01, ****p*< 0.001.


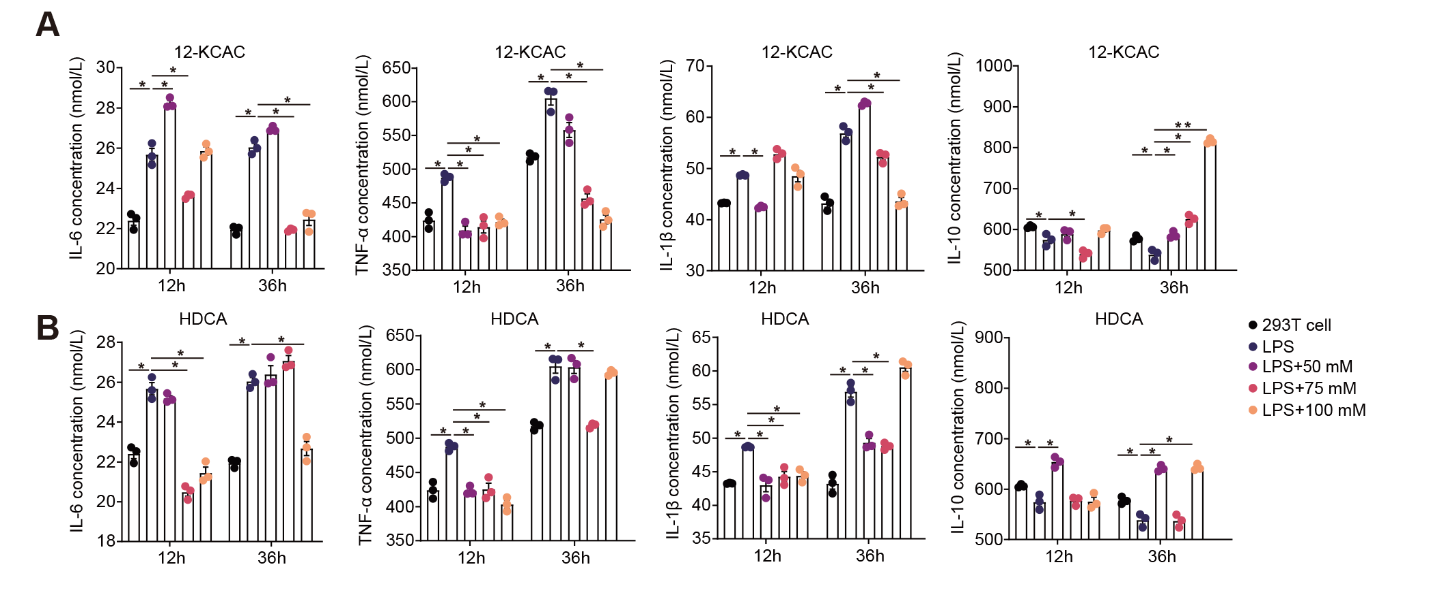


**Fig. S8. Changes in the concentrations of pro-inflammatory and anti-inflammatory cytokines in LPS-induced 293T inflammatory cell model upon treatment with 12-KCAC and HDCA.** Statistical significance was determined using one-way ANOVA, followed by Tukey test. **p* < 0.05, ***p* < 0.01, ****p*< 0.001.


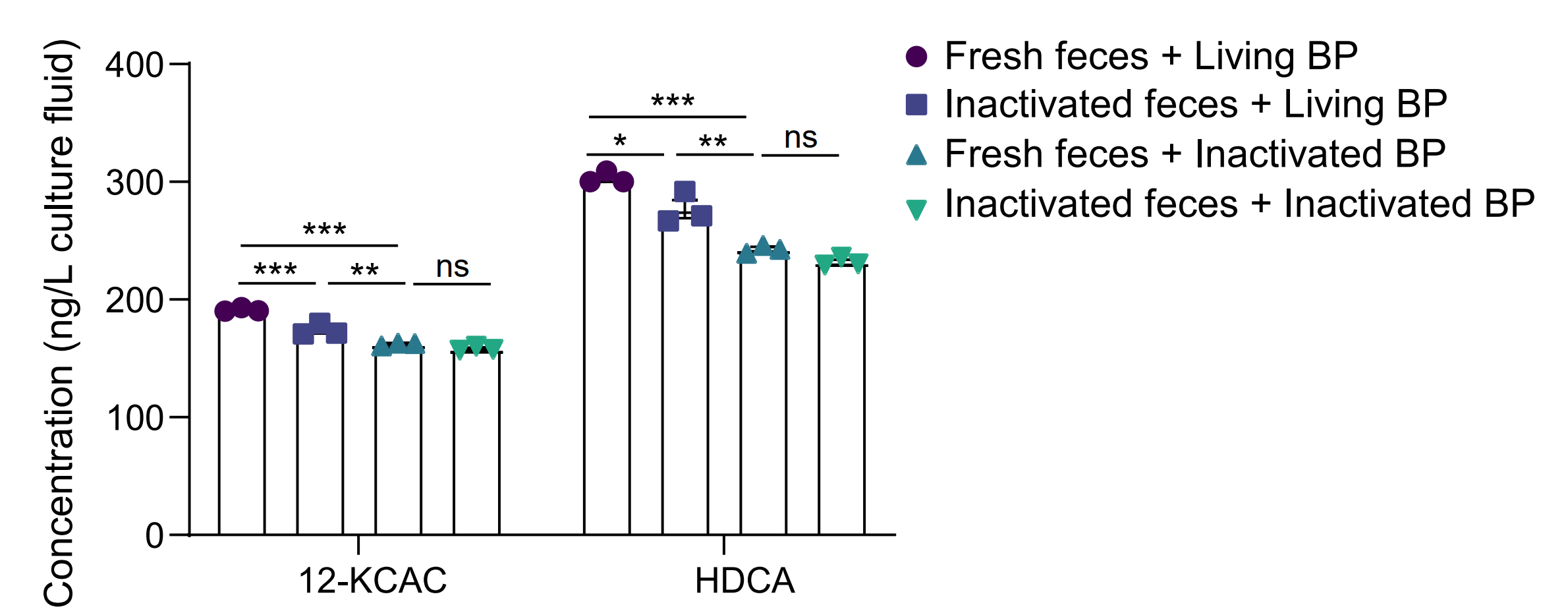


**Fig. S9.** The concentrations of 12-KCAC and HDCA in culture media treated with live and heat-inactivated B. pseudolongum RU224


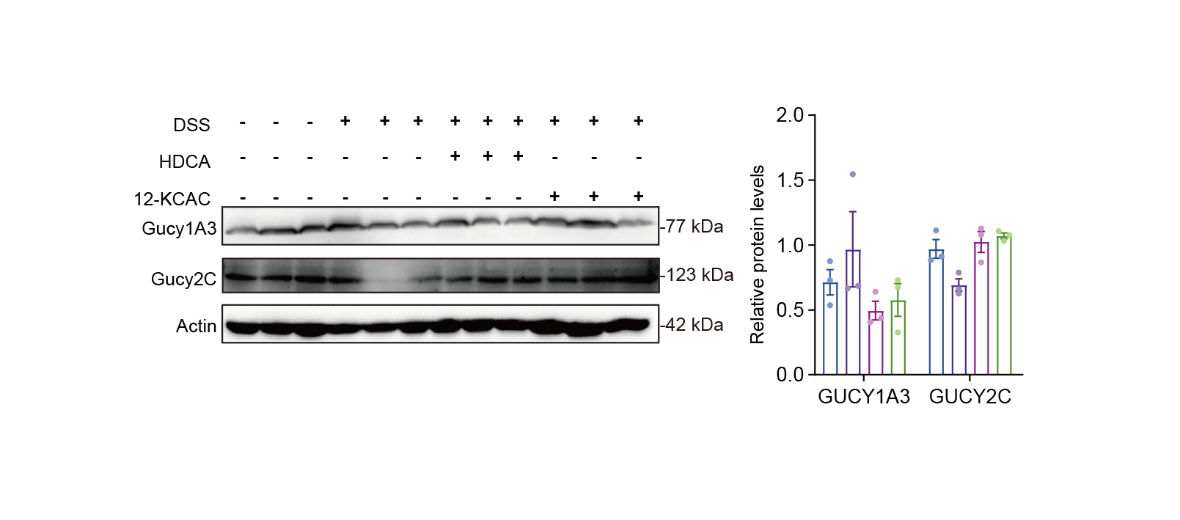


**Fig. S10.** Assessment of Gucy1A3 and Gucy2C levels in the colon of DSS-induced colitis mice treated with HDCA and 12-KCAC by western blot analysis, and quantification of protein band grayscale values using Image J.

Table S1. The dietary nutritional components of mice.

| **Nutritive Index** | **Content** |
| --- | --- |
| Moisture（g/kg） | ≤100 |
| Crude protein（g/kg） | ≥200 |
| Crude fat（g/kg） | ≥40 |
| Crude fibre（g/kg） | ≤50 |
| Crude ash（g/kg） | ≤80 |
| Calcium（g/kg） | 10～18 |
| Total phosphorus（g/kg） | 6～12 |
| Amino acid | |
| Lysine（g/kg） | ≥13.2 |
| Methionine + Cystine（g/kg） | ≥7.8 |
| Arginine（g/kg） | ≥11.0 |
| Histidine（g/kg） | ≥5.5 |
| Tryptophan（g/kg） | ≥2.5 |
| Phenylalanine + Tyrosine（g/kg） | ≥13.0 |
| Threonine（g/kg） | ≥8.8 |
| Leucine（g/kg） | ≥17.6 |
| Valine（g/kg） | ≥11.7 |
| Isoleucine（g/kg） | ≥10.3 |
| Minerals | |
| Magnesium（g/kg） | ≥2.0 |
| Kalium（g/kg） | ≥5.0 |
| Natrium（g/kg） | ≥2.0 |
| Iron（mg/kg） | ≥120 |
| Manganese（mg/kg） | ≥75 |
| Cuprum（mg/kg） | ≥10.0 |
| Zinc（mg/kg） | ≥30.0 |
| Iodine（mg/kg） | ≥0.5 |
| Selenium（mg/kg） | 0.1～0.2 |
| Energy supply ratio | |
| Protein（%） | 22.8 |
| Fat（%） | 13.8 |
| Carbohydrate（%） | 63.4 |
| Total heat（Kcal/kg） | 3656 |

Table S2. The primer sequences for quantitative real-time PCR analysis.

| **Gene Symbol** | **Forward（5’-3’）** | **Reverse（5’-3’）** | **Product Length** |
| --- | --- | --- | --- |
| *β-actin* | AGGGAAATCGTGCGTGACAT | GGAAAAGAGCCTCAGGGCAT | 172 |
| *TNF-α* | AGGCACTCCCCCAAAAGATG | TTTGCTACGACGTGGGCTAC | 250 |
| *IL-6* | AATAGTCCTTCCTACCCCAA | GCTTAGGCATAACGCACT | 167 |
| *IL-1β* | AACTGCACTACAGGCTCCGAGA | GCCACAGGTATTTTGTCGTTGCTT | 163 |
| *IL-10* | GCCGGGAAGACAATAACTGC | GCCTGGGGCATCACTTCTAC | 223 |
| *MUC2* | TGCTGACGAGTGGTTGGTGAATG | GATGAGGTGGCAGACAGGAGACA | 136 |
| *ZO-1* | GCCGCTAAGAGCACAGCAA | GCCCTCCTTTTAACACATCAGA | 172 |
| Claudin-1 | GGCCTTGGCTGTACCTTACC | GGAGCACCTTATCCCCGTTT | 109 |
| Occludin | TTGAAAGTCCACCTCCTTACAGA | CCGGATAAAAAGAGTACGCTGG | 129 |
